# Graphene inclusion controlling conductivity and gas sorption of metal–organic framework†

**DOI:** 10.1039/c8ra02439a

**Published:** 2018-04-16

**Authors:** Paolo Lamagni, Birgitte Lodberg Pedersen, Anita Godiksen, Susanne Mossin, Xin-Ming Hu, Steen Uttrup Pedersen, Kim Daasbjerg, Nina Lock

**Affiliations:** Carbon Dioxide Activation Center (CADIAC), Interdisciplinary Nanoscience Center (iNANO) and Dept. of Chemistry, Aarhus University Gustav Wieds Vej 14 DK-8000 Aarhus C Denmark nlock@inano.au.dk; Dept. of Chemistry, Technical University of Denmark Kemitorvet, DK-2800 Kgs. Lyngby Denmark

## Abstract

A general approach to prepare composite films of metal–organic frameworks and graphene has been developed. Films of copper(ii)-based HKUST-1 and HKUST-1/graphene composites were grown solvothermally on glassy carbon electrodes. The films were chemically tethered to the substrate by diazonium electrografting resulting in a large electrode coverage and good stability in solution for electrochemical studies. HKUST-1 has poor electrical conductivity, but we demonstrate that the addition of graphene to HKUST-1 partially restores the electrochemical activity of the electrodes. The enhanced activity, however, does not result in copper(ii) to copper(i) reduction in HKUST-1 at negative potentials. The materials were characterised in-depth: microscopy and grazing incidence X-ray diffraction demonstrate uniform films of crystalline HKUST-1, and Raman spectroscopy reveals that graphene is homogeneously distributed in the films. Gas sorption studies show that both HKUST-1 and HKUST-1/graphene have a large CO_2_/N_2_ selectivity, but the composite has a lower surface area and CO_2_ adsorption capacity in comparison with HKUST-1, while CO_2_ binds stronger to the composite at low pressures. Electron paramagnetic resonance spectroscopy reveals that both monomeric and dimeric copper units are present in the materials, and that the two materials behave differently upon hydration, *i.e.* HKUST-1/graphene reacts slower by interaction with water. The changed gas/vapour sorption properties and the improved electrochemical activity are two independent consequences of combining graphene with HKUST-1.

## Abstract 2

Changed electrochemical activity and CO_2_/H_2_O adsorption by graphene inclusion in Cu_3_(1,3,5-benzenetricarboxylate)_2_ and covalent tethering to glassy carbon electrodes.

## Graphical abstract

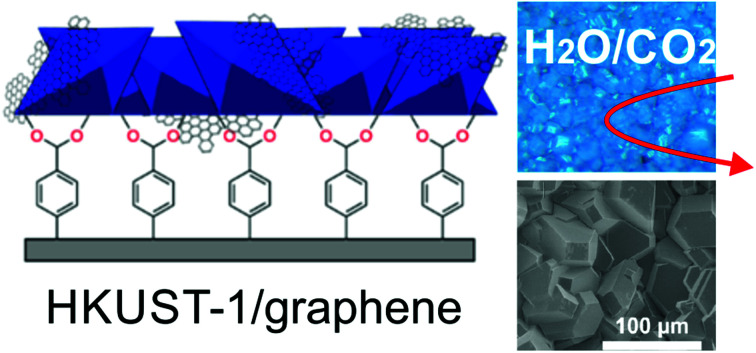

## Introduction

Metal–organic frameworks (MOFs) are porous coordination polymers in which metal centres are connected *via* linking molecular organic species. MOFs have attracted increasing attention over the past decades due to their various applications in gas storage,^1,2^ catalysis,^3–6^ luminescence,^7,8^ chemical sensing,^9,10^ water purification,^11^ and as scaffolds for guest molecules.^12^

MOFs are promising candidates for catalysis due to their metal centres. A number of experimental and computational^13^ studies have focused on using MOFs for catalytic reduction of CO_2_.^14^ Recent experimental examples include photocatalytic reduction of CO_2_ to formate,^15,16^ CO_2_ to CO,^17^ and CO_2_ to CO and methane.^18^ Moreover, electrocatalytic reduction of CO_2_ has been reported for numerous MOF systems to obtain *e.g.* methane^19^ and CO.^20^ A faradaic efficiency as high as 93(5)% has been achieved for the electrocatalytic reduction of CO_2_ to CO.^21^ One of the MOFs investigated for its catalytic properties, including CO_2_ reduction, is the compound HKUST-1, which is in focus in this study. HKUST-1 is a microporous cubic 3D network of copper(ii) dimers and 1,3,5-benzenetricarboxylate (BTC). The archetype Cu_3_(BTC)_2_ structure attains a so-called paddle-wheel structure with dimeric copper(ii) clusters as metal centres. HKUST-1 is one of the most studied MOFs, and the compound is even commercially available. The properties of HKUST-1 include storage of hydrogen,^22^ methane,^2,23^ methanol,^24^ oxygen,^25^ and CO_2_ (ref. 26 and 27) in addition to its negative thermal expansion properties^28^ and use in catalysis.^29–34^

HKUST-1 has been reported to be an active heterogeneous electrocatalyst for reduction reactions:^35^ significant studies include the reduction of CO_2_ to oxalic acid in organic medium as reported by Kumar *et al.*,^36^ and the conversion of CO_2_ to ethanol and methanol in water according to studies by Albo *et al.*^37^

In heterogeneous electrocatalysis, the active material is typically immobilised on a conducting electrode. However, most MOFs suffer poor conductivity, which hinders the electron transfer from the electrode to the catalytic metal sites and thus limits their efficiency. HKUST-1 in an electrical insulator,^38^ whereby thin coatings of this material may act as efficient blocking layers, limiting the use of HKUST-1 in electrochemical applications. Recently, large attention has been focused on improving the electrical and thermal conductivity of MOFs by combining a poorly conducting MOF with a conducting material such as carbon nanotubes^39^ or graphene derivatives.^40^ For example, composites of graphene oxide and HKUST-1 show an improved conductivity in addition to an increased CO_2_ adsorption capacity in comparison with bare HKUST-1.^41–43^

A widely applied procedure to immobilise heterogeneous electrocatalysts onto an electrode is drop casting. In this procedure a suspension of the catalyst in a Nafion-based ink is casted onto an electrode surface, *e.g.* a glassy carbon (GC) electrode, and a catalyst coating is formed on the surface after evaporation of the solvent. Despite the simplicity and many successful studies using this approach,^36,37,44^ it is not applicable for all types of materials due to limited adhesion strength, as the catalyst is not covalently tethered to the substrate. For example, solvothermally synthesised MOFs, including HKUST-1, often form large crystals with sizes of tens of microns. They easily detach from the electrode surface by immersion of the electrode into a solution. One method to form stable electrodes is by grafting the catalyst onto the electrode surface by means of covalent bonding. Hou *et al.* used a 4-carboxyphenyldiazonium salt to functionalise the surface of GC electrodes, which were subsequently immersed into a precursor solution to grow continuous defect-free films of MOF-5.^45^ Similarly, Balakrishnan *et al.* electrografted the same diazonium salt on GC plates to grow HKUST-1 films by means of solvothermal synthesis or microwave-assisted methods.^46^

In this study, we have developed a general and reliable method to prepare solution-resistant MOF-based composite coatings on glassy carbon substrates. Electrografting with diazonium salts and subsequent solvothermal synthesis were used to chemically tether HKUST-1 to glassy carbon substrates, similarly to the approach by Balakrishnan *et al.*, who focused on bare HKUST-1 and not on composites.^46^ Hybrid HKUST-1/graphene composites were prepared to form a bulk powder and film-coated electrodes with homogeneously distributed graphene flakes. Graphene was chosen due to its good electrical conductivity and charge transfer properties.^47–49^ The differences in material structure and properties between HKUST-1 and an HKUST-1/graphene composite with 8 wt% graphene are presented herein through detailed characterisation using X-ray diffraction (XRD), scanning electron microscopy (SEM), Raman mapping, electron paramagnetic resonance (EPR) spectroscopy, gas sorption studies, and electrochemical conductivity tests. Moreover, the electrocatalytic CO_2_ reduction properties of the bare MOF and composite films were investigated, and the stability of the films under working conditions was compared to that of drop casted films.

## Experimental

### Material synthesis

All chemicals were supplied by Sigma-Aldrich and used without further purification.

#### Preparation of graphene

Graphene was produced by mechanical exfoliation of graphite.^50^ In a conical flask 400 mL of *N*-methyl-2-pyrrolidone (NMP, HPLC grade) was added to 20 g of graphite (flake size < 20 μm). The head of a shear-mixer (ProScientific Pro SC-250, with a Pro's Standard 30 mm Generator head mounted) was dipped into the mixture and positioned approximately 2 mm from the bottom of the flask. The solution was blended for 3 h (rotation speed 10 000 rpm, corresponding to a shear rate of ∼18 500 s^−1^). The final solution was cooled down naturally to room temperature before centrifuging it for 20 minutes at 2700 rpm (Hettich Zentrifugen – Universal 16 A) to remove unexfoliated graphite. The supernatant contained the shear-exfoliated graphene, which was isolated by suction filtration and washed with ethanol. Finally, the graphene was dried overnight at 70 °C.

#### Functionalisation of glassy carbon (GC)

GC plates (10 × 10 × 1 mm^3^) were cleaned by sonication in acetone (HPLC grade), milliQ-H_2_O and pentane (HPLC grade), 10 minutes in each solvent, and subsequently dried in an argon stream.

The GC surface was modified by means of diazonium electrografting.^51–54^ A 0.1 M solution of tetrabutylammonium tetrafluoroborate (TBA·BF_4_) in acetonitrile (HPLC grade) was used as electrolyte, and 4-carboxyphenyldiazonium tetrafluoroborate was added to give a 2 mM solution. For the electrografting a three-electrode electrochemical setup was used; the GC plate was used as the working electrode, a Pt wire was used as counter electrode, while a Ag/AgI electrode was used as reference. Potentials recorded using the Ag/AgI reference electrode were converted to the saturated calomel electrode (SCE) reference by using the relation *E*_SCE_ = *E*_Ag/AgI_ – 0.380 V. Cyclic Voltammetry (CV) was used to graft the diazonium salt onto the GC surface by performing a single CV experiment in the range from +0.72 V to −0.88 V *vs.* SCE, using a scan rate of 10 V s^−1^.^54^ Finally, the functionalised GC plates were sonicated for 5 minutes in acetone (HPLC grade) and dried.

#### Synthesis of HKUST-1 and HKUST-1/graphene powders and films

HKUST-1 and HKUST-1/graphene composites were produced by solvothermal synthesis. Each synthesis produced a bulk powder in addition to a thin film on a pre-functionalised GC plate. A 50 mL Teflon lined stainless steel autoclave was used as reactor. The Teflon liner was modified to hold a GC plate vertically, approximately 17 mm from the bottom (Fig. S1†). The resulting inner volume of the Teflon liner was 44 mL.

We used a protocol by Balakrishnan *et al.*^46^ for synthesising HKUST-1 without graphene. A mixture of 1.107 g copper nitrate hemipentahydrate (Cu(NO_3_)_2_·5/2H_2_O, 4.6 mmol) and 0.533 g trimesic acid (H_3_BTC, 2.5 mmol) was dispersed in 24 mL of a 1 : 1 : 1 mixture (by volume) of *N*,*N*-dimethylformamide (DMF, ≥99%), deionised H_2_O and ethanol (≥99.8%). The mixture was sonicated for 5 minutes and transferred to an autoclave, which was sealed and heated at 85 °C for 19 h. After cooling to room temperature, the HKUST-1-covered GC plate was rinsed with ethanol, while the bulk powder was isolated by filtration and washed repeatedly with ethanol. Finally, the coated GC plate and the powder were dried overnight at 85 °C. Composite films (Fig. S2†) and powders of HKUST-1/graphene were obtained following the same procedure, with the only difference of adding 70 mg shear-exfoliated graphene to the MOF precursors before addition of the solvent. The added graphene corresponds to 8 wt% of the MOF product. The colour of the solution after the solvothermal reaction was black and opaque due to the dispersion of some graphene or graphite, which had not been incorporated into the MOF powder or film. After filtration the mother liquor was light blue, hinting that most of the dispersed graphene/graphite was isolated along with the bulk composite powder. We thus assume that the composite powder contained approximately 8 wt% graphene/graphite in total, while the amount of graphene in the composite films was estimated to be 6.4(1) wt% by XPS (Fig. S3 and Table S1†).

### Structural and physical characterisation

#### X-ray diffraction (XRD)

Powder X-ray diffraction data (PXRD) were measured on a Smartlab diffractometer from Rigaku using Cu Kα_1_ radiation (*λ* = 1.54056 Å). Diffracted intensities were collected in the angular range 5–60° on a D/TEX Ultra 256 strip detector. Data on the bulk powders were measured in Bragg–Brentano geometry. The crystalline films were analysed using grazing incidence X-ray diffraction (GI-XRD) on the same diffractometer using convergent beam optics and a 1° grazing angle.

#### Optical microscopy

Optical images were acquired using an Olympus SZX16 stereomicroscope allowing magnifications up to ×11.5.

#### Scanning electron microscopy (SEM)

SEM images of HKUST-1 and HKUST-1/graphene (powders and films) were obtained on a FEI-Nova Nano SEM 600 scanning electron microscope under low vacuum conditions (60 Pa water vapour). The powders were immobilised on sticky carbon tape.

#### Raman spectroscopy

Bulk powders and films were characterised using a Renishaw InVia Reflex Raman Microscope/Spectrometer. The instrument is equipped with an Ar-ion laser (excitation wavelength of *λ* = 514 nm; *I*_max_ = 100 mW) and a 1200 lines per mm grating monochromator.

#### X-ray photoelectron spectroscopy (XPS)

XPS analysis was performed on a Kratos Axis Ultra-DLD spectrometer using Al Kα X-rays (150 W). Survey spectra were measured in constant analyser energy (CAE) mode at a pass energy of 160 eV, and the analysis area was 300 × 700 μm^2^. Charge compensation was achieved by using an electron flood gun.

#### Gas adsorption

Gas adsorption and desorption isotherms (N_2_ at 77 K and 273 K, and CO_2_ at 273 K and 295 K) were measured on an Autosorp iQ from Quantachrome to determine the specific surface area and porosity of the powders, in addition to investigating their gas adsorption capacity. The heat of adsorption of CO_2_ to the powders was extracted from the CO_2_ isotherms. Bulk powders of HKUST-1 and HKUST-1/graphene composites were activated by outgassing for 10 hours at 230 °C under dynamic vacuum prior to the measurements.

#### Electron paramagnetic resonance (EPR) spectroscopy

*In situ* EPR measurements were performed on the bulk powders with a Bruker EMX X-band spectrometer using the setup described by Godiksen *et al.*^55^ The powders were pressed into pellets at relatively low pressure, crushed and sieved in order to obtain a uniform particle size distribution where all parts of the sample would interact equally with a gas flow. Samples of 20 mg powder (150–300 μm) were immobilised with quartz wool in tubes with an inner diameter of 4 mm and exposed for 1 h to a helium flow of 200 mL min^−1^, corresponding to a gas hourly space velocity (GHSV) of 400 000 h^−1^, at temperatures up to 230 °C to dehydrate the samples. The temperature was then decreased to room temperature, at which the samples were firstly exposed to a flow of CO_2_ and then to a static atmosphere of CO_2_. Finally, the samples were flushed with helium for 5 minutes and exposed to water vapour (approximately 1.2 vol%) in a helium flow at room temperature (200 mL min^−1^). Each individual EPR spectrum was obtained over approximately 45 s, and the field was swept for additionally 16 s giving a time resolution of 61 s. The EPR parameters were: frequency 9.45 GHz, microwave power 6.5 mW, modulation 100 kHz, modulation amplitude 8 G and field sweep 120 to 500 mT. The quality of the tuning was continuously checked, and the frequency was adjusted slightly if necessary. Cavity *Q*-values were constant during the *in situ* investigations.

*Ex situ* investigations were performed in 4 mm suprasil quartz EPR tubes at room temperature and at 77 K. The *ex situ* EPR spectra (averaged over three sweeps) were compared to solid copper(ii) standard samples prepared by mortaring a known amount of CuSO_4_·5H_2_O in K_2_SO_4_.

All spectra were background corrected by subtracting the spectrum of an empty tube. The flow was either pure helium (AGA, 99.995%) or pure CO_2_ (AGA, 99.95%). For hydration half the helium gas flow was equilibrated with water by bubbling it through an autoclave with water.

### Electrochemical characterisation

Experiments were performed using a CH Instrument 601D or an AutoLab potentiostat. Before each measurement argon or CO_2_ was bubbled through the solution for at least 10 minutes.

#### Electrocatalytic tests

The electrocatalytic activity was investigated by CV with a scan rate of 0.050 V s^−1^. An electrochemical H-cell was used with a film coated GC-plate as working electrode and a Ag/AgCl reference electrode in one compartment (*E*_SCE_ = *E*_Ag/AgCl_ – 0.045 V), while a Pt mesh (counter electrode) was placed in the other compartment. The supporting electrolyte solution was 0.01 M TBA·BF_4_ in acetonitrile.

#### Electron transfer properties

Electron transfer properties of MOF and composite films on GC substrates were investigated in a three-electrode electrochemical cell by adding ferrocene (Fc) to an electrolyte solution of 0.01 M TBA·BF_4_ in argon saturated acetonitrile to give a 2 mM Fc solution. The potential was scanned from +0.12 V to +0.62 V *vs.* SCE, and backwards, with a sweep rate of 0.1 V s^−1^. The sample under investigation was used as working electrode, while a Pt wire was used as counter electrode. A mask was attached to the working electrode in order to have the same circular area (*Ø* = 2.74 mm) exposed in all measurements.

## Results and discussion

### HKUST-1 and composite film formation

Initially, powders of solvothermally synthesised HKUST-1 and HKUST-1/graphene were suspended in a Nafion ink and drop casted onto a GC disc electrode surface by following a typical procedure.^44^ It was observed that the films started peeling off the GC electrode when dipped into an electrolyte solution in acetonitrile, and they detached completely after just one or two CV cycles (Fig. S4†). Hence, the HKUST-1 electrodes produced by drop casting were not useful for practical applications due to their limited stability.

**Fig. 1:**
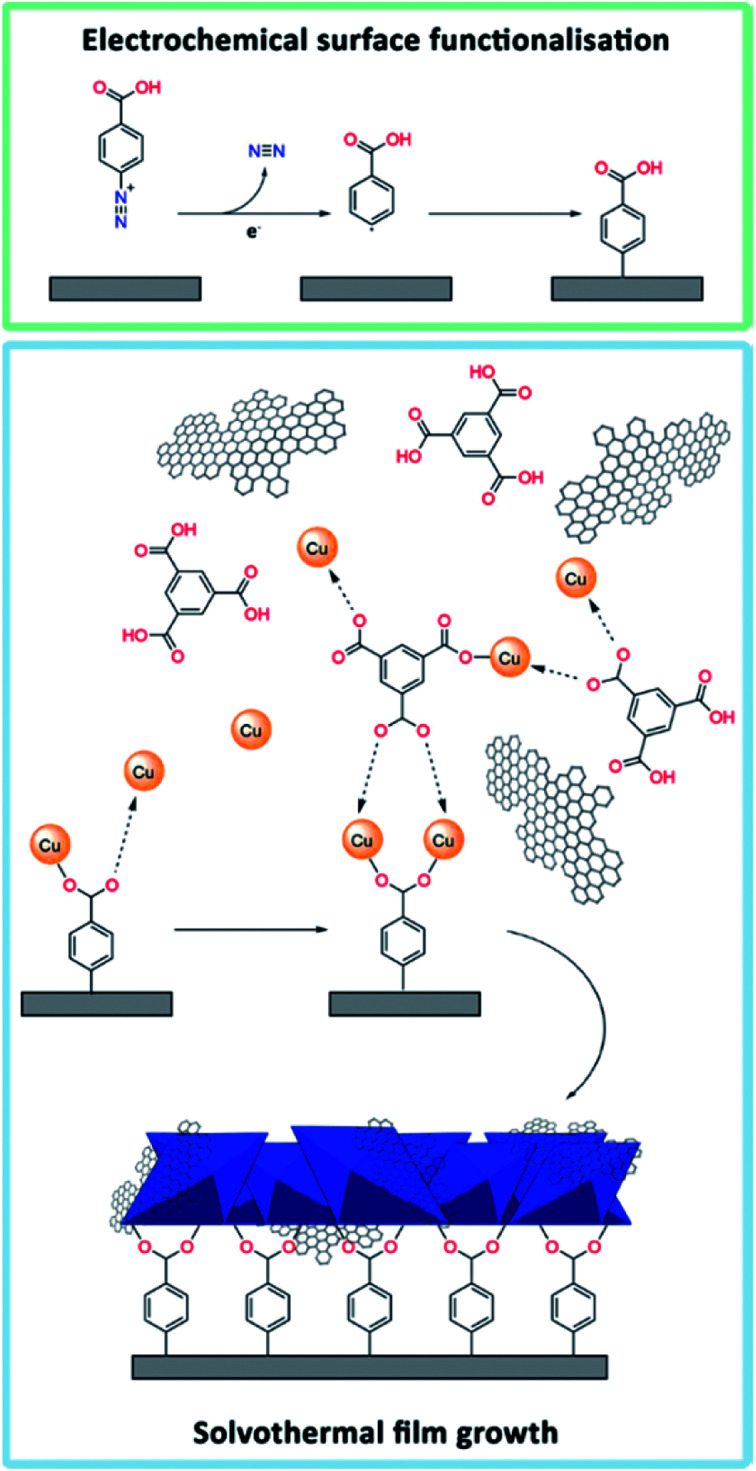
Graphical representation of the HKUST-1/graphene film formation: (top) electrografting of 4-carboxyphenyl diazonium salt onto a GC substrate. (Bottom) solvothermal synthesis of an HKUST-1/graphene composite film, which is covalently tethered to the functionalised GC plate. The carboxylates on GC coordinate to Cu^2+^ (charges not shown) and thereby act as HKUST-1 nucleation sites. The graphene sheets, precursors, and HKUST-1 crystals (blue octahedra) are not shown on scale.

With the aim of increasing the stability of MOF-based electrodes, attention was focused on covalent tethering of the MOF onto the substrate. The first attempts of growing HKUST-1 directly on a bare GC plate did not lead to any film formation (Fig. S5†). Therefore, we took advantage of pre-functionalising the GC surface by electrografting using 4-carboxyphenyldiazonium tetrafluoroborate (Fig. 1). This diazonium salt resembles the linker in HKUST-1 (*i.e.* trimesic acid), and the carboxylic acid groups were expected to serve as nucleation sites to promote the formation of HKUST-1 directly onto the GC substrate. The electrografting was performed by CV, using fast potential sweep rates (10 V s^−1^) in order to form thin layers of carboxyphenyl moieties. Reduction of the diazonium salt was indicated by the appearance of a broad reduction peak at approximately −0.57 V *vs.* SCE in the respective cyclic voltammogram (Fig. S6†). Our approach partly differs from the procedure by Balakrishnan *et al.*, who used multiple scans at lower sweep rates and half of the diazonium concentration in comparison with this study. The procedure by Balakrishnan *et al.*^46^ led to the formation of thicker films.

In order to grow an HKUST-1 film onto the diazonium functionalised GC substrate, the GC plate was attached to a modified Teflon autoclave liner (Fig. S1†) and immersed into a solution of the HKUST-1 precursors. Moreover, aiming to improve the conductive properties of the films by exploiting the properties of graphene,^47–49^ HKUST-1/graphene films were synthesised by adding shear-exfoliated graphene to the MOF precursors. The solvothermal process produced a blue crystalline HKUST-1 film (Fig. S2†), which was covalently attached to the GC plate, and a bulk powder. To our knowledge, no similar procedure has previously been reported to form stabile HKUST-1/graphene composites on electrodes.

### Physical characterisation of films and powders

**Fig. 2:**
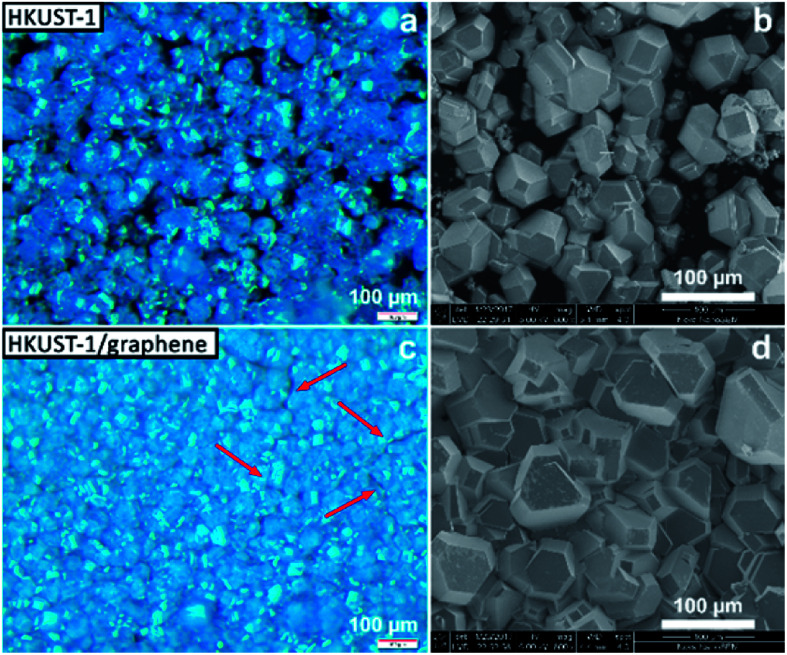
Optical microscopy (left) and SEM (right) images of films of HKUST-1 (top) and HKUST-1/graphene (bottom). The HKUST-1 crystals form clusters on the surface of GC, leaving several areas uncoated, while the addition of graphene leads to complete coverage of the substrate. The red arrows indicate small cracks in the composites, possibly caused by mechanical stress in the films.

Several techniques were used to investigate the quality of the synthesised films, as well as to verify the homogeneity of HKUST-1 and graphene in the composites. Optical microscopy was used to study the coverage and uniformity of the films. Both HKUST-1 and HKUST-1/graphene formed crystals with sizes of approximately 50–100 μm on the GC surface (Fig. 2). Several pinholes are observable in the bare MOF films, while the graphene composites generally form denser films with nearly complete coverage, *i.e.* the addition of graphene seems to promote nucleation on the substrate surface. The higher crystal density of the composite films introduced some mechanical strain resulting in small cracks in the films (Fig. 2c). Scanning electron microscopy (SEM) images (Fig. 2 and S7†) show good agreement with optical microscopy with respect to film coverage and crystallite sizes. The SEM images of the composite film (Fig. 2d and S7†) display micron-sized bright features at some crystal faces; these features are possibly graphene flakes.

**Fig. 3:**
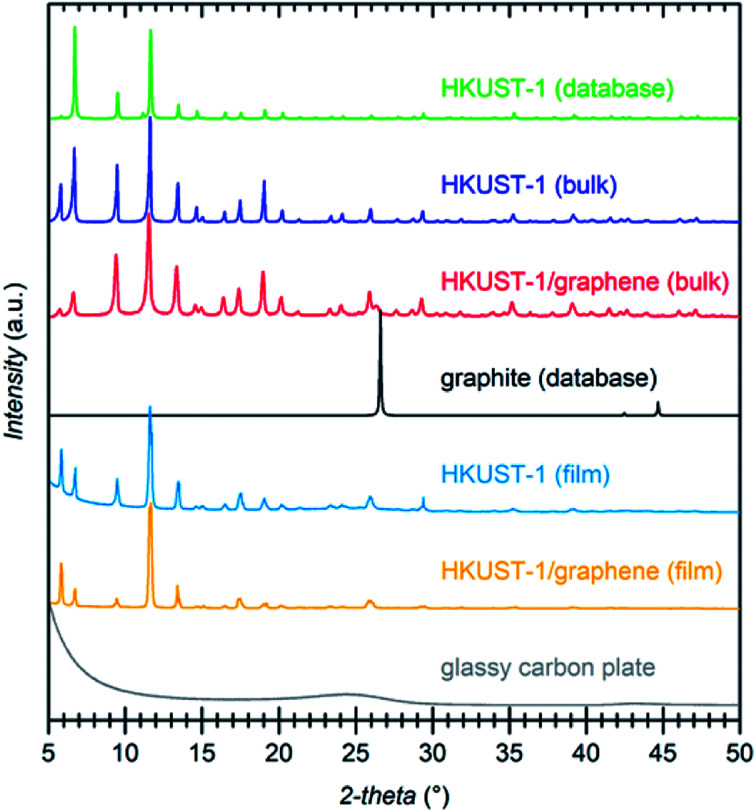
PXRD patterns of: HKUST-1/graphene composite powder (red), HKUST-1 powder (blue), and a calculated reference diffraction pattern for HKUST-1 (CCDC 697917) (green). HKUST-1 is phase pure in the bare MOF sample. In the composite, graphite is present in addition to HKUST-1 as indicated by the peak at 26.3° corresponding to the (002) reflection of graphite (black, calculated, AMCSD 0011247). GI-XRD patterns of the MOF (light blue) and composite (orange) films reveal crystalline HKUST-1. The diffraction pattern of an uncoated glassy carbon plate is shown in grey.

The composition of the materials was studied by X-ray diffraction (XRD) and Raman spectroscopy. XRD data were collected on the bulk powders and on the films (Fig. 3). PXRD data of both HKUST-1 and HKUST-1/graphene powders overall match the HKUST-1 reference pattern in terms of peak positions and relative peak intensities. However, there are some differences: (1) the relative intensities of the two peaks at low 2-theta angles strongly differ relative to the intensities of the peaks at 9° and 12°. The relative intensities of the low-angle peaks depend on the degree of solvation inside the HKUST-1 pores (Fig. S8†). (2) The composite powder shows one additional diffraction peak at 26.3°, which corresponds to the (002) graphite/multi-layer graphene reflection representing the stacking of sp^2^ carbon layers. It is likely that graphite/multi-layer graphene was present in the bulk powder as a consequence of graphene stacking during the solvothermal treatment and subsequent collection by filtration (see Experimental section). However, presence of multi-layer graphene/graphite does not exclude simultaneous presence of few-layer or even single-layer graphene in the powders. GI-XRD data collected on the film samples confirm the formation of HKUST-1 crystallites on the GC plates. The relative intensities differ from those of HKUST-1 powders, but preferred orientation of crystallites in the films may be present. It should be noticed that diffraction peaks of graphite/multi-layer graphene are absent in the composite film diffraction pattern. This excludes the presence of graphite in the films, but does not contribute any information on the inclusion of few-layer or single-layer graphene. As verified by Raman spectroscopy studies of the films (see below) and XPS, a carbon material was incorporated into the films (Fig. S9†).^50^

**Fig. 4:**
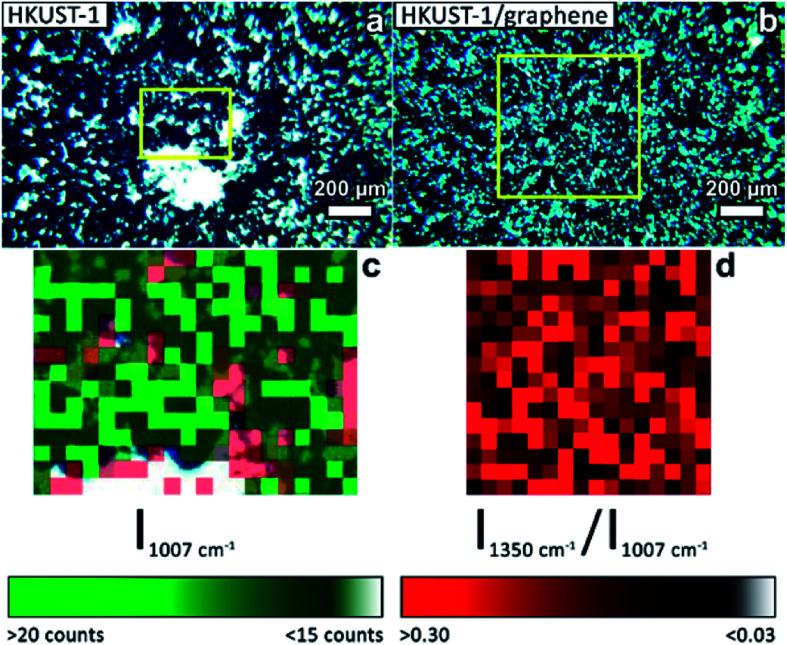
Optical images acquired on the Raman microscope of (a) an HKUST-1 film and (b) a composite HKUST-1/graphene film. (c) Overlap of two Raman maps of an HKUST-1 film (pixel size: 20 × 20 μm^2^) corresponding to the area inside the yellow square in (a). Green pixels are used to map MOF-covered areas: bright green areas are indicative of a high MOF concentration; the green scale bar represents the intensity of the most intense Raman signal of HKUST-1 (1007 cm^−1^). Red pixels represent areas where GC peaks dominate the Raman spectra. The two colour maps match the regions where the MOF crystals are observed in the optical image (blue crystals, green pixels), and GC is exposed at uncoated areas (bright areas in (a), red pixels in (c)). (d) Raman map of an HKUST-1/graphene composite film (pixel size: 40 × 40 μm^2^) corresponding to the area inside the yellow square in (b). The red map highlights graphene containing areas. The red scale bar represents the intensity ratio between the D peak of the carbon phase (1350 cm^−1^) and the most intense signal of HKUST-1 (1007 cm^−1^).

The presence of graphene in the composite films was proven by Raman mapping of the film-covered GC substrates. Area-resolved Raman spectra were collected on films of HKUST-1 and HKUST-1/graphene (Fig. 4) and compared to spectra of pure GC, graphene and HKUST-1 powder (Fig. S9†). Fig. 4a and b display images of HKUST-1 and HKUST-1/graphene composite films as acquired on the Raman microscope. Dark blue HKUST-1 crystals are observable in addition to white areas which are spots of uncovered GC reflecting light in the measurement chamber. Raman maps were overlapped with the optical images of the HKUST-1 (Fig. 4c) and HKUST-1/graphene (Fig. 4d) films. The intensity of a Raman spectrum corresponding to one pixel is low, and the maps should not be used for quantification but as an overview of the present phases. For the bare HKUST-1 sample (Fig. 4c) areas with a high concentration of HKUST-1 are shown in bright green, whereas dark green spots represent areas where the MOF phase can still be detected, despite not being in perfect focus due to the micrometre-scale roughness of the film. The red map in Fig. 4c represents areas where the Raman signal of GC is more intense than that of HKUST-1, *i.e.* red pixels represent uncoated GC. Pixels representing inconclusive spectra due to a high noise level were left transparent (see also Fig. S9a†). The green and red maps are complementary, and they are in good agreement with the optical image in Fig. 4a, *i.e.* the green Raman map overlaps the HKUST-1 crystals on the optical image. Fig. 4d shows the Raman maps of an HKUST-1/graphene composite film. Here, brighter red points highlight areas with a higher concentration of graphene, whereas black spots represent areas with low or no graphene content. Overall, the Raman map shows a high degree of homogeneity of HKUST-1 and graphene at the micrometre scale. Graphene and graphite may generally be distinguished by Raman spectroscopy. The 2D Raman peak of graphene is symmetric, while the 2D peak of graphite is split and has a shoulder located on the low-wavenumber side of the peak.^56,57^ In the spectrum of the composite film (Fig. S9e†) a broad peak with low intensity (compared to the HKUST-1 Raman spectrum) is observed at ∼2700 cm^−1^. Presence of a peak at 2690 cm^−1^ would correspond to graphene, while the 2D peak of graphite would be centred around 2725 cm^−1^.^56^ Our Raman spectroscopy data clearly confirms the presence of a carbon material in the composite, but the intensity and resolution of the Raman data do not allow distinguishing between graphene and graphite. However, GI-XRD showed no indication of graphite in the composite film, and we propose graphene is present. Even though it would basically be impossible to distinguish graphene from GC in the spectra of the composite films, the spectra are unlikely to contain strong signals from GC, as the SEM images (Fig. 2) reveal that no or few pinholes were present in the composite films.

### Gas sorption of HKUST-1 and HKUST-1/graphene composites

**Fig. 5:**
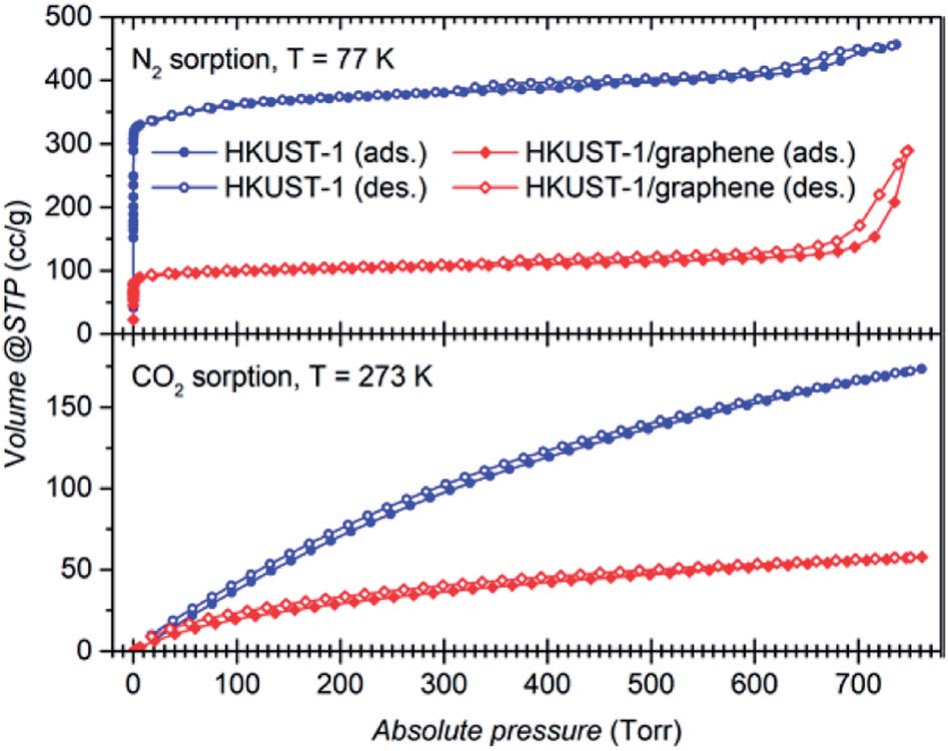
(Top) Adsorption (filled symbols) and desorption (open symbols) curves at 77 K for N_2_ over bulk HKUST-1 (blue) and over the composite (red). (Bottom) adsorption and desorption curves for CO_2_ at 273 K of the same samples. The isotherms show higher adsorption capacity of the bare MOF compared to HKUST-1/graphene.

Gas sorption studies were conducted on the bulk powders to investigate whether the presence of graphene plays a role on the gas uptake properties of the material. Full adsorption and desorption isotherms were collected using N_2_ at 77 K and 273 K, and CO_2_ at 273 K and 295 K (Fig. 5 and S10a and b†). Gas sorption at 273 K reveals a large selective uptake of CO_2_ over N_2_ in both samples (Fig. S10a and b†). The selectivity was quantified to a factor of approximately 15 and 9, respectively, for HKUST-1 and the composite at 760 torr (Fig. S10c and d†). In other words, in the pressure range from 100 torr to 760 torr, parent HKUST-1 has selectivity for CO_2_ over N_2_ which is approximately 1.5 times higher than that of HKUST-1/graphene; we are unable to explain this difference in selectivity. Nevertheless, the composite binds CO_2_ stronger at small amounts (*q*) of adsorbed CO_2_, as quantified by its larger heat of adsorption (Δ*H*_ads_) of approximately −29 kJ mol^−1^ for the composite and −23 kJ mol^−1^ for HKUST-1 at *q* = 0.5 mmol g^−1^ (Fig. S10e†). The heat of adsorption of the composite is even pressure dependent, while being constant for the MOF. The larger heat of adsorption of the composite may be due to a stronger interaction between hydrophobic graphene sheets and non-polar CO_2_ molecules or differences in the microstructure at the HKUST-1/graphene interface (see below).

The shape of the isotherms collected for N_2_ at 77 K (Fig. 5) gives information about the porosity of the materials.^58^ HKUST-1 presents a curve with shape of a type I isotherm, representative of microporous systems (pores smaller than 2 nm), mixed with a type IV isotherm at higher pressures, indicating presence of mesopores as also suggested by the material pore size distribution (Fig. S10f and g†). However, from the crystal structure it is known that crystalline HKUST-1 does not contain mesopores. Defects in the crystal lattice may locally result in a changed pore structure. For comparison, HKUST-1/graphene has an isotherm resembling type I in combination with type II at high relative pressures. A type II isotherm is indicative of macropores or non-porous powders. Correspondingly, the BET specific surface area drops from 1423 m^2^ g^−1^ for HKUST-1, which is consistent with literature reports,^22^ to 393 m^2^ g^−1^ for HKUST-1/graphene. As HKUST-1 forms microsized crystals of a rigid well-defined scaffold, it is unlikely that the microporous structure of the MOF would be altered by the addition of graphene to HKUST-1, and the HKUST-1 structure was confirmed by PXRD. However, addition of graphene might change the HKUST-1/graphene interfaces and result in additional gas sorption sites, *e.g.* pockets and pores formed due to non-ideal contact between the graphene sheets and the MOF facets. However, there is no other experimental evidence of macropore formation (resulting in type II isotherm behaviour), but presence of mesopores in the composite powder is hinted (Fig. S10f†). Changes in the microstructure of the material might explain the differences in the heat of adsorption between HKUST-1 and the composite, but it does not account for the large differences in the BET and micropore surface areas. We hypothesise that the graphene flakes might partly wrap the outer facets of the MOF crystals, thereby partly hindering the diffusion of N_2_ and CO_2_ into the HKUST-1 micropores. This could lead to local non-porous behaviour, which could also give rise to a type II isotherm and decreased adsorption capacity. Alternatively, the type II isotherm may be ascribed to the presence of graphite in the composite powder, but it seems unlikely that this small impurity accounts for the pronounced difference in gas sorption behaviour between HKUST-1 and HKUST-1/graphene.

Increased BET surface areas were reported by the addition of graphene oxide/graphite oxide (GO) to HKUST-1 and thereby contrasts our results.^41–43^ Instead, our observations regarding surface area changes and differences in the heat of adsorption are in good agreement with findings for composites of HKUST-1 and carbon nanotubes.^39^

### Electron paramagnetic resonance spectroscopy

EPR is a useful tool for determining the identity of paramagnetic copper sites. The time-resolved development in the EPR spectrum was monitored on powders of HKUST-1 and HKUST-1/graphene while (i) dehydrating the materials at a temperature corresponding to the outgassing temperature during the gas sorption studies (230 °C), (ii) exposure to CO_2_ and (iii) rehydration at room temperature.

Recent literature describes the result of applying EPR to HKUST-1, and several types of copper(ii) sites are discussed:^59–63^ (1) monomeric copper such as [Cu(H_2_O)_*n*_]^2+^ (*n* = 5 or 6) captured in the cavities. These have spin quantum number *S* = 1/2; (2) paddle-wheel units consisting of two copper centres bridged by four carboxylate groups. The copper atoms couple antiferromagnetically giving a diamagnetic *S* = 0 ground state and an *S* = 1 excited state which will be populated at higher temperatures; (3) paddle-wheel units that have interacted with water changing the Cu–Cu interaction giving either more or less strongly interacting Cu centres. As a consequence of these different types of copper(ii) in HKUST-1, the EPR signal is composed of several contributions. The signals are also influenced by the high concentration of paramagnetic centres in the samples. Together this makes it exceedingly difficult to assess the contribution from each type of copper species.

In this manuscript, we have taken a different approach to follow dehydration, CO_2_ adsorption and rehydration by *in situ* EPR. This gives dual information: (1) the spectra themselves that may have identifying features. Interaction is often revealed between the unpaired electron and the copper nuclei, which has a nuclear spin of 3/2 in both copper isotopes (^63^Cu, 61% and ^65^Cu, 39%). (2) The development in the intensity of the EPR signal (obtained as the double integral of the measured first derivative spectra) with temperature and treatment with adsorbates. The intensity gives information on the overall development in the population of EPR active copper centres.

**Fig. 6:**
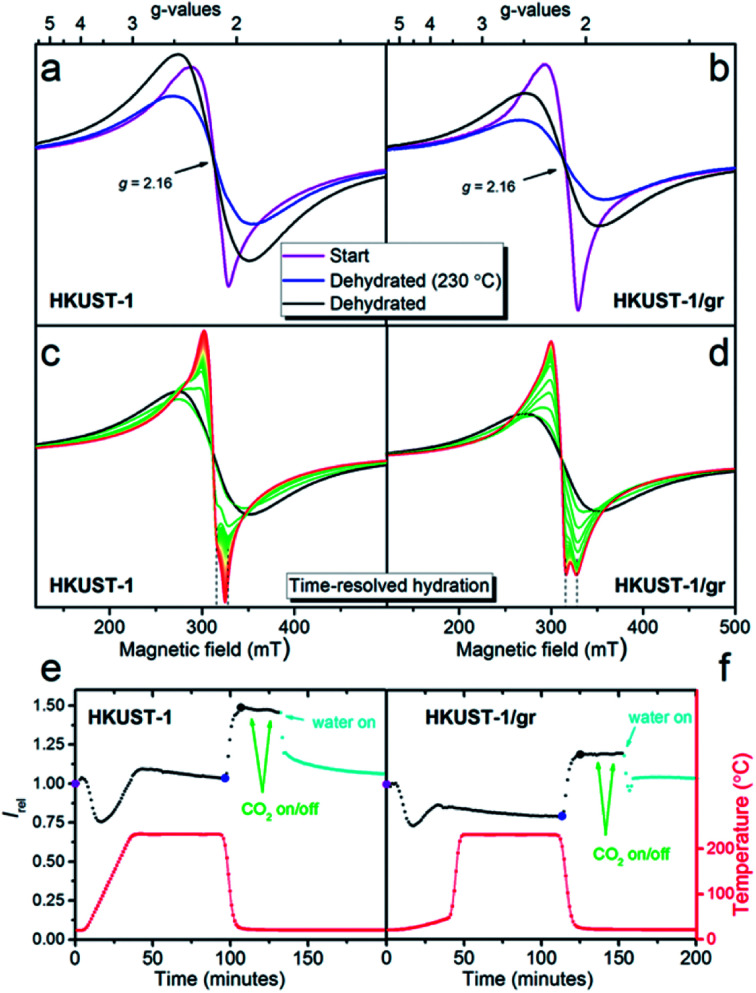
Room temperature X-band first derivative EPR spectra of HKUST-1 (a) and HKUST-1/graphene (b). Spectra at ambient conditions at time zero are given in purple. Spectra after exposure to a dry He flow during the temperature ramp to 230 °C followed by a constant flow for 1 hour at this temperature are given in blue. Spectra after cooling to room temperature, still under flow, are given in black. (c) and (d) Time-resolved EPR spectra of the same two samples during exposure of the dehydrated samples to H_2_O (approximately 1.2 vol%) in a He flow. Spectra are approximately 1 minute apart. (e) and (f) EPR signal intensity (the double integral), relative to the EPR intensity at the starting point, is given as a function of time. The points corresponding to the spectra given in (a) and (b) are shown with larger dots (purple, blue and black). The points corresponding to the spectra in (c) and (d) are given in teal. From the large blue dots to the large black dots the intensity increases by 47(3)%. The temperatures measured right next to the sample for each spectrum are given as the red curve.

Initially the EPR spectra of both samples were compared at room temperature (*ex situ*) to reference *S* = 1/2 copper(ii) samples in order to quantify the observed signal (Fig. S11†). Under the assumption that the observed signal is due to non-interacting *S* = 1/2 copper(ii) centres obeying the Curie law (monomers), the EPR signal corresponds to a copper content of 5.2(5) wt% for HKUST-1 and 4.5(5) wt% for HKUST-1/graphene. This can be compared to a theoretical total amount of 29 wt% copper in pure hydrated HKUST-1 (Cu_3_(BTC)_2_·3H_2_O). As outlined above this assumption is not valid since copper is distributed over several sites. Dimeric species follow another intensity profile. In addition, the concentration of paramagnetic centres in HKUST-1 is very high and long-range interactions are present. However, the calculation still serves as an indication of how much signal is missing due to magnetic interactions. The spectral features at room temperature are broad, not completely symmetric and are centred at a *g*-factor, *i.e.* the dimensionless magnetic moment, equal to 2.16 (Fig. 6a and b). Copper paddle-wheel dimers give broad symmetric spectra at room temperature whereas copper monomers give a much narrower and more anisotropic spectrum.^62^ We conclude that both monomers and dimers are present here. There is no evidence of the distinct anisotropic *S* = 1 dimer type signal^59,62^ on our HKUST-1 samples at room temperature or at 77 K. However, PXRD clearly reveals HKUST-1 (containing dimeric paddle-wheels), and we conclude that due to the high concentration of paramagnetic species the exchange narrowing of the *S* = 1 signal has resulted in complete conversion to an isotropic signal.^62^

During the *in situ* studies, the time-resolved EPR spectrum was monitored during the whole procedure of hydration, CO_2_ exposure and rehydration. An overview of the experiment giving the doubly integrated EPR spectra as a function of time and atmosphere for each sample is shown in Fig. 6e and f. Selected individual spectra are shown in Fig. 6a–d.

Upon dehydration, the spectrum becomes broad and completely symmetric (Fig. 6a and b). The total intensity obtained by double integration (Fig. 6e and f) first drops moderately, then increases and stabilizes at a value, which is slightly higher than the start value for HKUST-1 and lower than the start value for HKUST-1/graphene. After the temperature decrease to room temperature, the spectra do not change shape. The intensity increases to a value which is 47(3)% higher at 22 °C compared to the value at 230 °C for both samples. This intensity gain is between the two limiting situations, *i.e.* 70% for pure monomeric copper and 10% for pure dimeric copper (Fig. S12 and details in the ESI†). Therefore, again both monomeric and dimeric copper species are suggested to be present in both samples.

Exposure to CO_2_ at ambient temperature and pressure did not have a discernible effect upon the EPR spectrum or intensity of either sample. This is perhaps not surprising since such a weak perturbation of the copper environment would be lost in the line width.^63^

Exposure to water vapour in flow at room temperature results in a less intense EPR spectrum. The intensity levels off at approximately the same value as the starting point for both samples. This corresponds to a loss of intensity of 31% for HKUST-1 and 17% for HKUST-1/graphene compared to the dehydrated state. If the process could be described in a simple way as dimeric paddle-wheel units breaking up upon hydration (making two magnetically isolated *S* = 1/2 copper(ii) monomers) the double integral of the EPR spectrum should have increased upon this treatment. This is clearly not the case. Todaro *et al.* hypothesise that paddle-wheel dimers, which are EPR silent due to an even stronger coupling, could be formed upon hydration of the material.^59^

Interestingly, the spectra of the two samples show different development upon exposure to water in the flow system (Fig. 6c and d). The EPR spectrum of HKUST-1 narrows after only 2 minutes exposure to water, and features which must be due to two different types of copper EPR signals are observed, in accordance with literature.^62^ With prolonged exposure to water, the features of the narrowest signal disappear and the other line shifts slowly to lower fields. For HKUST-1/graphene the development to a narrow spectrum with features belonging to two different Cu species is much slower (15 minutes) and the change in spectrum stops at this stage; no further development or loss of intensity are observed even after more than an hour. One explanation for this behaviour could be that graphene protects the EPR active copper centres towards further hydration and possible degradation in excess water.^59,62^ This would be in good agreement with the gas sorption analysis, in which we propose that graphene flakes partly wrap around HKUST-1 crystals and partly block the access to the pores. Thus, the access of water to the copper sites may be hindered by hydrophobic graphene causing slower kinetics of the hydration. The suggested presence of copper(ii) monomers in the structure may be ascribed to crystal defects.

### Electrochemical characterisation and catalysis performance test

**Fig. 7:**
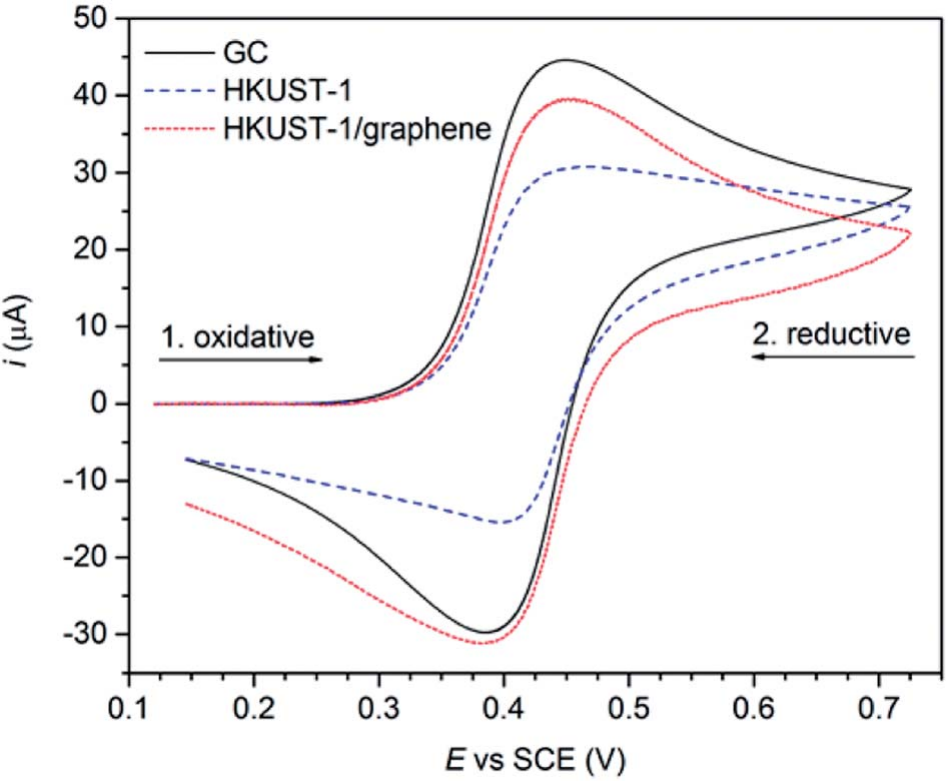
CV measurements (recorded using a Ag/AgI reference electrode in 0.01 M TBA·BF_4_/acetonitrile, sweep rate: 0.1 V s^−1^) on a blank GC plate (black), a HKUST-1-covered GC plate (blue) and a composite film on GC (red) using 2 mM Fc as redox probe. These curves were obtained applying baseline removal and iR compensation to the original voltammograms (see Fig. S13†).

The effect of graphene on the conductivity of the HKUST-1 film was studied using cyclic voltammetry. Ferrocene (Fc, 2 mM) was employed as an outer-sphere redox probe to test the electron transfer across the film. Uncoated GC was used as a reference, showing typical oxidative and reductive waves of Fc centred at the standard potential of +0.418 V *vs.* SCE (Fig. 7). With HKUST-1 coated on the GC electrode a flat voltammogram of Fc would be expected, as HKUST-1 is insulating.^38^ Moreover, the MOF films are so thick (∼50 μm) that tunnelling through the film is unexpected to occur. In fact, the cyclic voltammogram recorded using HKUST-1-coated GC shows both anodic and cathodic currents as high as two thirds of those for the bare GC. This can be explained by the fact that the HKUST-1 coverage on GC is not complete, leaving some pinholes uncovered (Fig. 2 and S13†). Still, the blocking effect of the MOF layer is indicated by the shape of the voltammogram peak tails; rather than pure planar diffusion the voltammogram partly resembles a steady-state regime which is often seen for arrays of ultramicroelectrodes. For comparison, the HKUST-1/graphene composite film shows improved conductivity, based on the shape of the cyclic voltammogram, *i.e.* the diffusion-limited regime is restored in the peak tails. The current response of the composite film appears to be increasing with peak values close to those of the bare GC electrode. Considering the more compact coating of the HKUST-1/graphene composite film as shown in Fig. 2, the electron transport between the GC electrode and Fc in solution has improved by inclusion of graphene.

Importantly, the films were proven to be stable under electrochemical conditions (Fig. S14b and c†) showing that the diazonium grafting is a good approach to attach MOFs onto electrode materials. No material loss was observed after several CV cycles (Fig. S14a†). We speculate that using grafted carboxyphenyl groups as nucleation sites for MOF growth may be applied to a large variety of MOFs. For example, Hou *et al.* demonstrated the use of diazonium grafting to synthesise films of cubic MOF-5 onto glassy carbon electrodes by introducing 4-carboxy-phenyl groups as covalent linkers on the electrode surface.^45^ Ideally, the same method could also be extended to other conductive substrates, such as gold and stainless steel, due to the non-specificity of electrografting and the mild reaction conditions of the solvothermal approach.

Even though the electron transfer properties improved by combining HKUST-1 with graphene, the voltammograms in Fig. 7 do not prove an enhanced electron transfer to the copper(ii) sites in HKUST-1. Therefore, catalytic tests using HKUST-1 and composite films as electrocatalysts were performed by following procedures by: (1) Kumar *et al.* converting CO_2_ to oxalic acid in a DMF-based electrolyte solution,^36^ (2) Albo *et al.* who reduced CO_2_ to a mixture of methanol and ethanol in an aqueous electrolyte solution,^37^ and (3) Qin *et al.* who used HKUST-1 for electrocatalytic hydrogen production in aqueous sulphuric acid.^35^ Unfortunately, we were not able to reproduce the literature results, neither by using the covalently attached films (HKUST-1 or composite), nor by drop casting of the electrocatalyst onto GC electrode surfaces. We observed a complete degradation of HKUST-1 when soaked in the aqueous KHCO_3_ electrolyte solution used by Albo *et al.*^37^ and in 0.5 M H_2_SO_4(aq)_ as reported by Qin *et al.* (Fig. S15 and S16†).^35^ As an example of an electrocatalytic CO_2_ reduction test, Fig. S17† displays cyclic voltammograms conducted in argon- and CO_2_-saturated solutions of TBA·BF_4_ in acetonitrile. Only an insignificant increase in current was observed when CV studies were performed in a CO_2_-saturated solution, compared to an argon-saturated solution.

The addition of graphene did not lead to improved electron transfer from the electrode to the copper(ii) sites in HKUST-1. We suspect that the limited electron transport within the crystals is caused by their large size, and that electron transport between GC and the electrolyte solution is mainly occurring along the crystal–graphene–crystal interfaces rather than through the crystals. We suggest that smaller crystals (*e.g.* sub-micrometre sized particles) could promote an efficient charge transfer to the catalytic sites, which would possibly activate them for reduction reactions. If the graphene flakes partly hinder the pathways of CO_2_ and water molecules to the copper-sites in the HKUST-1/graphene composites as suggested by the gas sorption studies and EPR spectroscopy, respectively, this may be another reason why the catalytic properties are not improved for the composite. Another reason could be due to the electronic properties of undoped graphene, which has no electronic states close the Fermi level. This may result in a slow electron transfer between GC and graphene.

## Conclusion

We have demonstrated that stable films of crystalline HKUST-1 and HKUST-1/graphene composites containing 6.4(1) wt% graphene can be synthesised on GC. Rapid pre-functionalisation of a GC substrate by electrografting of a diazonium salt resulted in the formation a thin carboxyphenyl film, which was covalently bound to the electrode. By subsequent solvothermal synthesis films of HKUST-1 and HKUST-1/graphene composites with a thickness of tens of microns were formed in addition to the corresponding bulk powders. Raman spectroscopy mapping suggests that graphene is distributed uniformly in the films. GI-XRD reveals HKUST-1 as the only crystalline phase of the films, while bulk composite powders reveal presence of a graphite impurity.

Investigation by EPR spectroscopy shows the samples behave similarly during *in situ* dehydration, but a difference is observed in the subsequent rehydration. We speculate this is due to a change in the hydrophilicity of the material by integration of hydrophobic graphene layers. The observed lower degree of interaction between the composite and water might also cause changed interactions between the composite and non-polar CO_2_ molecules. For example, gas sorption studies propose CO_2_ to bind stronger to the composite than HKUST-1 at low pressures. Moreover, gas adsorption reveals that the CO_2_ adsorption capacity is lower for the composite powder than for the bare HKUST-1. This can be ascribed to the larger BET surface area of HKUST-1 in comparison with the composite powder, which may be due to a partial blocking of the micropores by graphene coverage of the crystals surface.

The beneficial role of adding graphene to HKUST-1 was proven. It favours the formation of uniform and complete films, and the covalently tethered films were shown to be stable in electrolyte solution during multiple CV cycles in contrast to drop casted films of the same material. Moreover, the electron transfer across the blocking MOF layer was restored in the composite films. Since no improved electrocatalytic activity was shown for the composite, electron transfer from conducting graphene to the copper(ii) sites in HKUST-1 did not occur. This can most likely be ascribed to the micrometer sized MOF crystallites and its poor electrical conductivity.

Overall, we have demonstrated the formation of stabile MOFs and composites as films and powders, and we suggest the approach can be applied to various metal–organic compounds on a variety of substrates (such as gold or aluminium) for electrocatalysis or other applications requiring stabile surface coatings.

## Conflicts of interest

There are no conflicts to declare.

## Supplementary Material

RA-008-C8RA02439A-s001
