# Supplementary material for: Graphene inclusion controlling conductivity and gas sorption of metal–organic framework
Source: RSC Adv. 2018 Apr 16;8(25):13921–32. doi: 10.1039/c8ra02439a (PMC9079844; doi:10.1039/c8ra02439a)
Supplement: RA-008-C8RA02439A-s001 [file RA-008-C8RA02439A-s001.pdf]

Electronic Supplementary Material (ESI) for RSC Advances.
This journal is © The Royal Society of Chemistry 2018

ELECTRONIC SUPPLEMENTARY INFORMATION:

Graphene inclusion controlling conductivity and gas sorption of metal-organic framework

Paolo Lamagni,[a] Birgitte Lodberg Pedersen,[a] Susanne Mossin,[b] Anita Godiksen,[b] Xin-Ming Hu,[a] Steen Uttrup Pedersen,[a] Kim Daasbjerg,[a] Nina Lock[a]*

[a] Carbon Dioxide Activation Center (CADIAC), Interdisciplinary Nanoscience Center (iNANO) and Dept. of Chemistry, Aarhus University, Gustav Wieds Vej 14, DK-8000 Aarhus C, Denmark

[b] Dept. of Chem., Technical University of Denmark, Kemitorvet, DK-2800 Kgs. Lyngby, Denmark

*Corresponding author: nlock@inano.au.dk

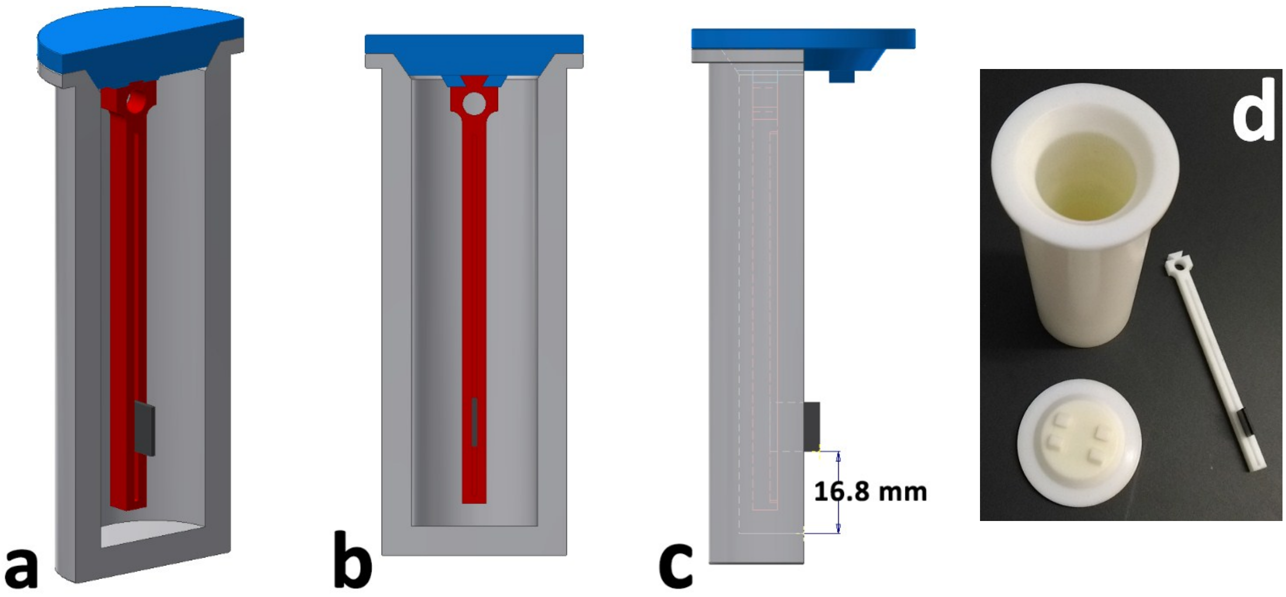

Fig. S1. (a)-(c) Design of the modified Teflon autoclave liner. The lid (blue) was modified in order to clamp a rod (red) for holding GC plates (dark grey). Substrates can be positioned at different heights, but a distance of 16.8 mm from the bottom of the vessel was used consistently in our experiments. (d) Photo of the home-made Teflon parts used for the solvothermal synthesis.

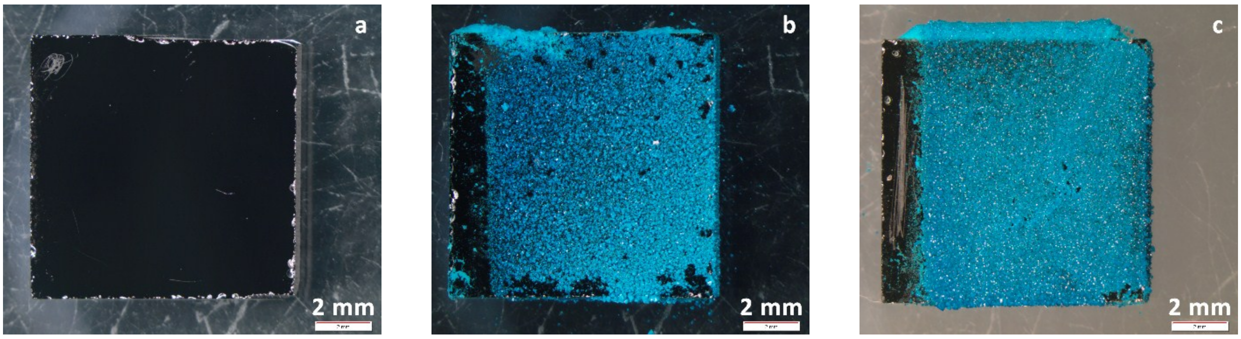

Fig. S2. Optical microscopy images of (a) a blank GC substrate, (b) an HKUST-1 film, and (c) an HKUST-1/graphene composite film. The uncoated left edge corresponds to the area where the plate was clamped into the Teflon holder while performing the synthesis, *i.e.* it was not in contact with the precursor solution. The composite film appears to be more homogeneous and to have a more complete coverage. The defects in the top right and bottom right corners are due to manipulation of the plates with tweezers. The top edge on the photo corresponds to the edge pointing upwards in the solvothermal reactor. Here, crystals have accumulated from the solution due to gravity effects. The software *ImageJ* was used to quantify the areas of exposed/uncoated GC to 16(3)% based on 5 different HKUST-1-coated plates.

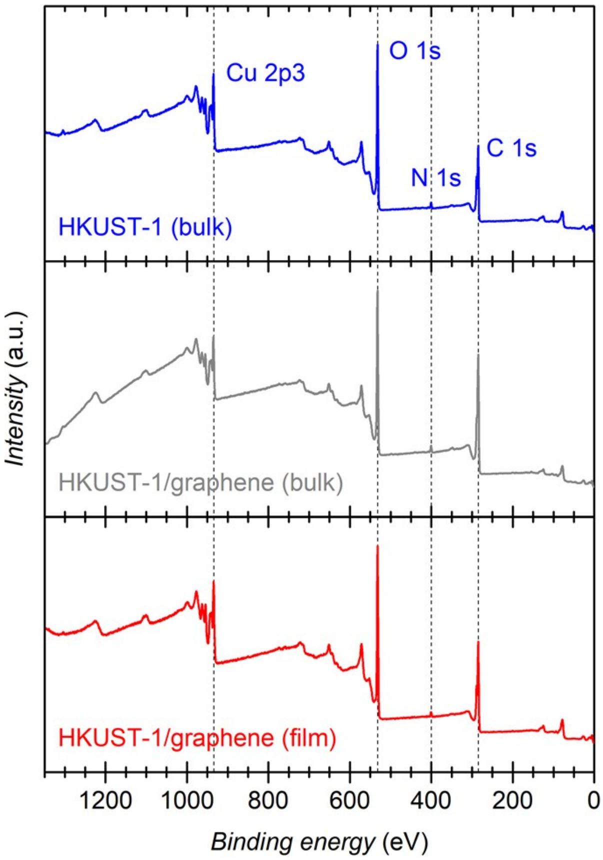

Fig. S3. XPS spectra of bulk HKUST-1 powder (blue), of a composite powder (grey), and of an HKUST-1/graphene film (red), showing the presence of Cu, C, O and N in all the samples. The small N content is probably due to residual DMF trapped in the HKUST-1 cavities.

Sample	Cu (at%)	C (at%)	O (at%)	N (at%)	ΔC (wt%)
**HKUST-1 (theoretical)**	9	55	36	-	-
**HKUST-1 (bulk)**	7.3(4)	57.6(3)	33.2(5)	1.84(6)	2.7(7) ǂ
**HKUST-1/graphene (bulk)**	4.69(6)	68.5(3)	25.3(3)	1.59(7)	11.0(2) •
**HKUST-1/graphene (film)**	5.3(7)	61(4)	33(3)	1.16(8)	6.39(8) •

Table S1. Elemental composition of the samples (H not included) based on XPS. The extra content of C, ΔC, which is ascribed to the inclusion of graphene (•), was calculated relative to the experimental MOF composition. In the case of bulk HKUST-1, the small additional C content (ǂ) compared to the theoretical composition may be ascribed to residual solvent trapped in the pores. The extra carbon contents in the composite bulk powder (11.0(2) wt%) and film (6.39(8) wt%) are close to the 8 wt% added in the synthesis, but the contributions from graphene and residual solvent in the pores may not easily be distinguished, and a graphene content of 6.4(1) wt% in the composite film (reported in the manuscript) is most likely overestimated. The surface composition of C and Cu of the composites may not be fully representative of the sample; the slight deficiency of C in the composite film may be due to presence of graphene in the film, which is not probed by surface sensitive XPS, or the low values for Cu may indicate graphene surface coverage of the composite powder and film.

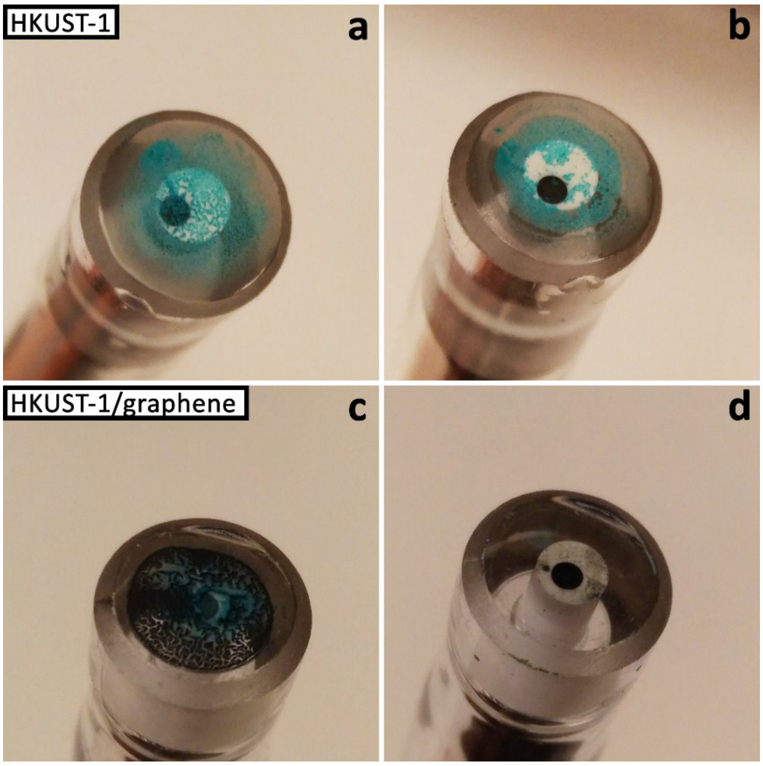

Fig. S4. Glassy carbon (GC) disc electrodes (Ø = 1 mm) coated with: (a)-(b) HKUST-1 and (c)-(d) HKUST-1/graphene by drop casting. (a) and (c) show the disc electrodes before running electrochemical tests. (b) and (d) show the same electrodes after one CV cycle. The films were damaged or completely lost during these tests.

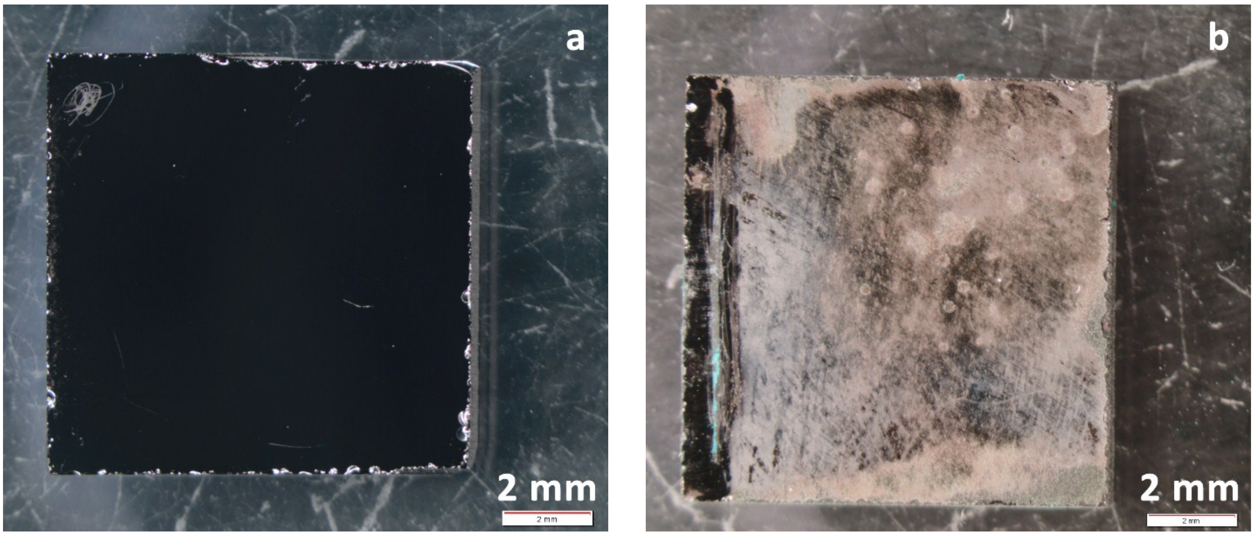

Fig. S5. Comparison between (a) a bare GC plate and (b) the first attempt of HKUST-1 film formation directly on unfunctionalised GC. A HKUST-1 synthesis protocol without DMF was used in the initial studies. No successful MOF growth was observed under these conditions.

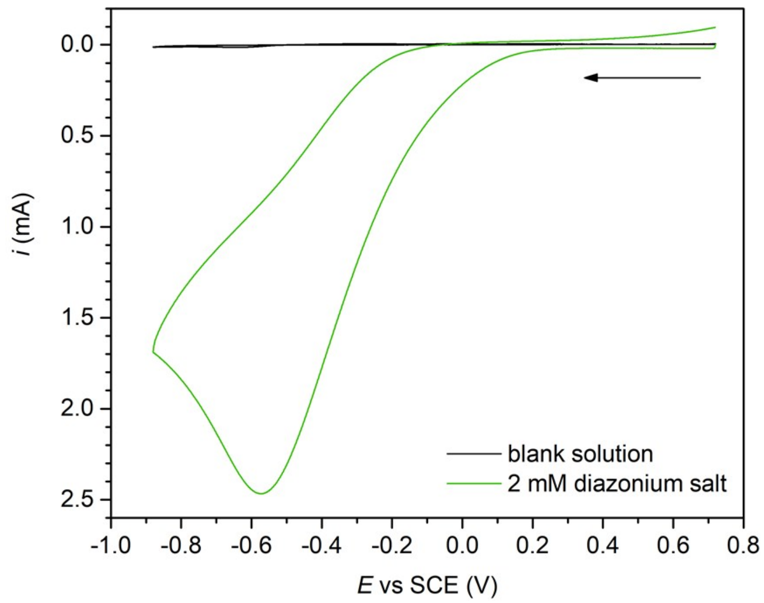

Fig. S6. Cyclic voltammogram showing the electrografting of 4-carboxyphenyldiazonium on GC using a diazonium salt as precursor. In absence of the diazonium salt, no reaction occurs at the electrode surface (black). After addition of the diazonium salt, a reduction peak centred at –0.57 V vs. SCE indicates successful functionalisation of the electrode (green). The voltammogram was recorded in 0.1 M $TBA\cdot BF_4$/acetonitrile at a sweep rate of 10 V s^{-1}, and the concentration of 4-carboxyphenyldiazonium tetrafluoroborate was 2 mM.

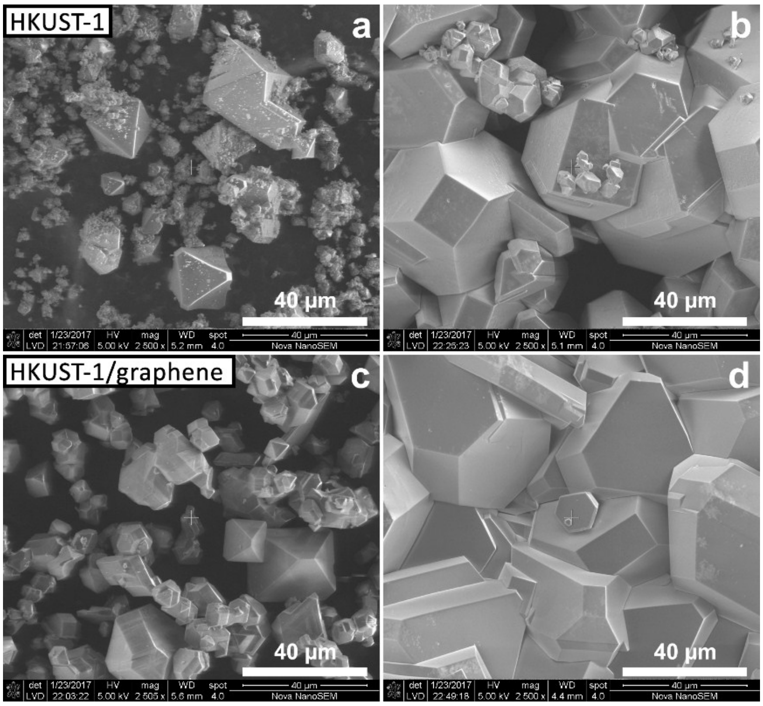

Fig. S7. SEM images of (a) HKUST-1 bulk powder, (b) HKUST-1 film, (c) HKUST-1/graphene bulk powder and (d) HKUST-1/graphene film. The crystals in the films are generally larger than the bulk powder crystals. HKUST-1 (a)-(b) forms octahedra, truncated cubes and cuboctahedra with dimensions of 10–50 μm. Several smaller crystallites with sizes of a few microns are also observable in the powder. The HKUST-1/graphene samples (c)-(d) show crystals with similar sizes and morphologies. The coverage of the HKUST-1 film (b) is not perfect as it contains several pinholes. For comparison, HKUST-1/graphene forms a denser film (d). The scale bar is the same on all four SEM images (40 μm).

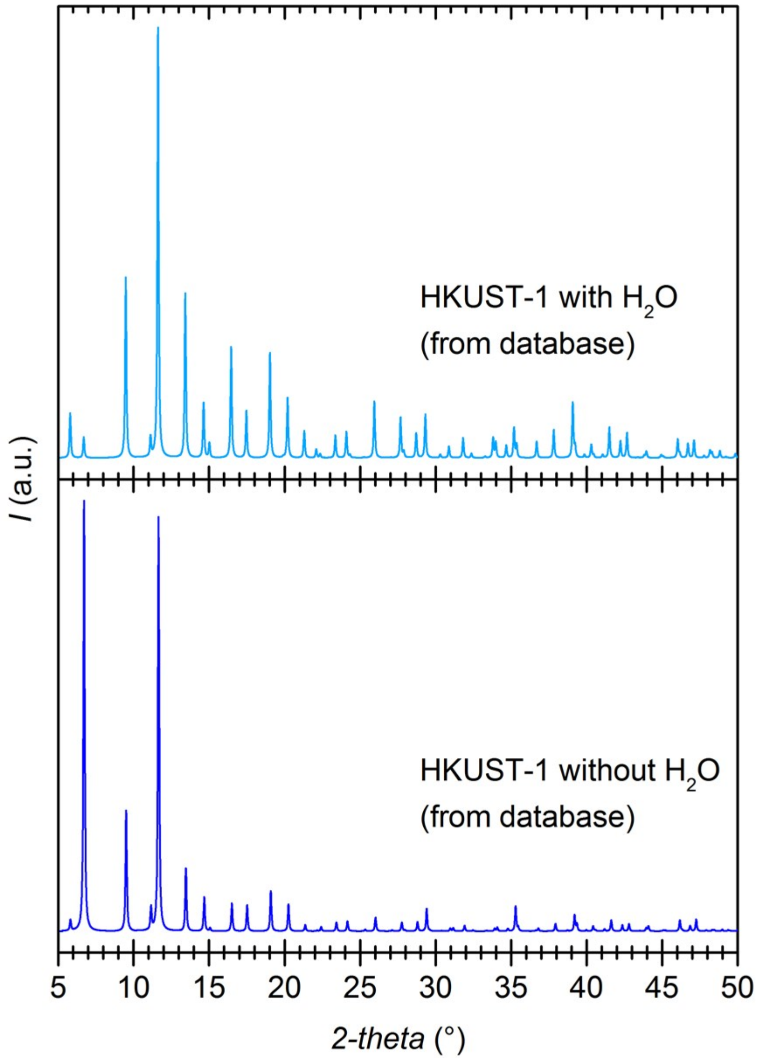

Fig. S8. Calculated PXRD patterns of solvent-free HKUST-1 (*bottom*) and H_2O-solvated HKUST-1 (*top*), based on database entries. The relative diffraction peak intensities change depending on the degree of hydration of the coordinatively unsaturated Cu(II) sites in HKUST-1, especially at low 2ϑ angles. Solvent-free HKUST-1 reference: CCDC 697917; water-containing HKUST-1 reference: CCDC 827934.

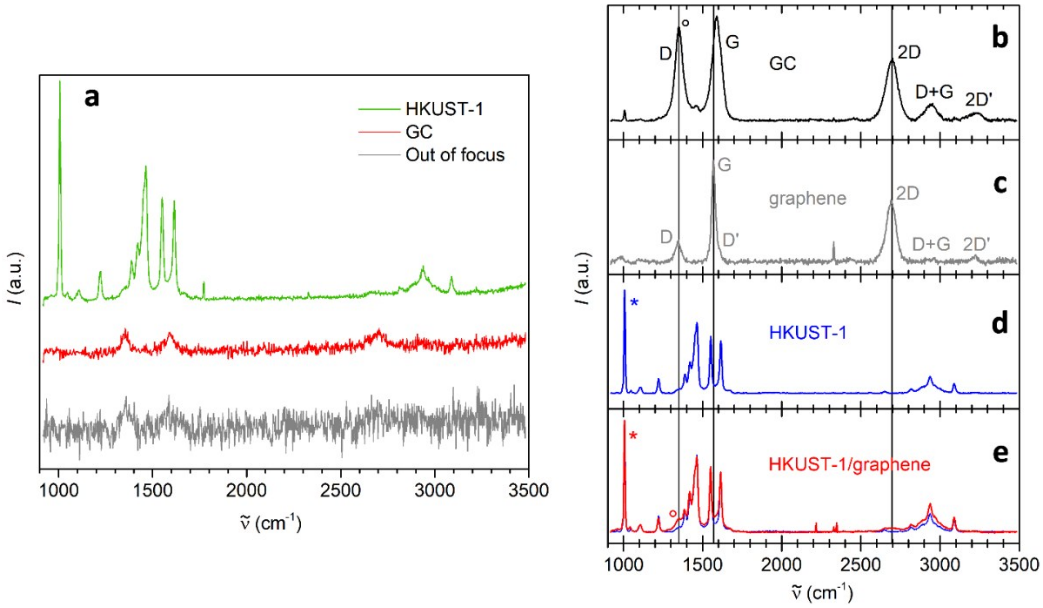

Fig. S9. (a) Raman spectra corresponding to the maps of HKUST-1 and GC in the manuscript Fig. 4c are plotted as averaged spectra, *i.e.* spectra from green (red) pixels are represented as the green (red) curve. Spectra from pixels with high noise (transparent pixels in Fig. 4c) are shown in grey. The intensity scale is common for the three spectra, and the average spectrum of GC (red) has a higher signal-to-noise ratio than the HKUST-1 spectrum (green), due to the substrate plane being out of focus compared to the top of the MOF films. The spectra clearly reveal HKUST-1, while features identifying GC are just visible.

The spectra on the right-hand side refer to Fig. 4b. Raman spectra of phase pure electrode materials: GC (b), graphene (c) and HKUST-1 (d). The characteristic peaks for the carbon-based materials are shown: The G peak represents the presence of sp^2-hybridised C, whereas the D and D′ peaks (and their overtones and combinations) refer to defects in the honeycomb structure of graphene and to sp^3-hybridised C atoms. Detailed descriptions of these signals can be found in literature.[1-3] The weak and sharp signal around 2330 cm^{-1} corresponds to gaseous N_2 in the measurement chamber. The Raman peaks marked with * and ° are those used for generation of the Raman maps presented in the manuscript Fig. 4. In particular, the green map (Fig. 4c) was obtained by mapping the intensity of the MOF peak at 1007 cm^{-1}, whereas the red maps (Fig. 4d) were drawn by mapping the intensity ratio of the peak at 1350 cm^{-1} of the carbonaceous species over that at 1007 cm^{-1} of HKUST-1. A comparison between the average Raman spectra of HKUST-1/graphene (film, red) and of HKUST-1 (bulk, blue) have also been added (e). A small increase in the Raman intensity (red curve) around the positions of the D, G and 2D peaks hint successful incorporation of a carbon material into the composite films.

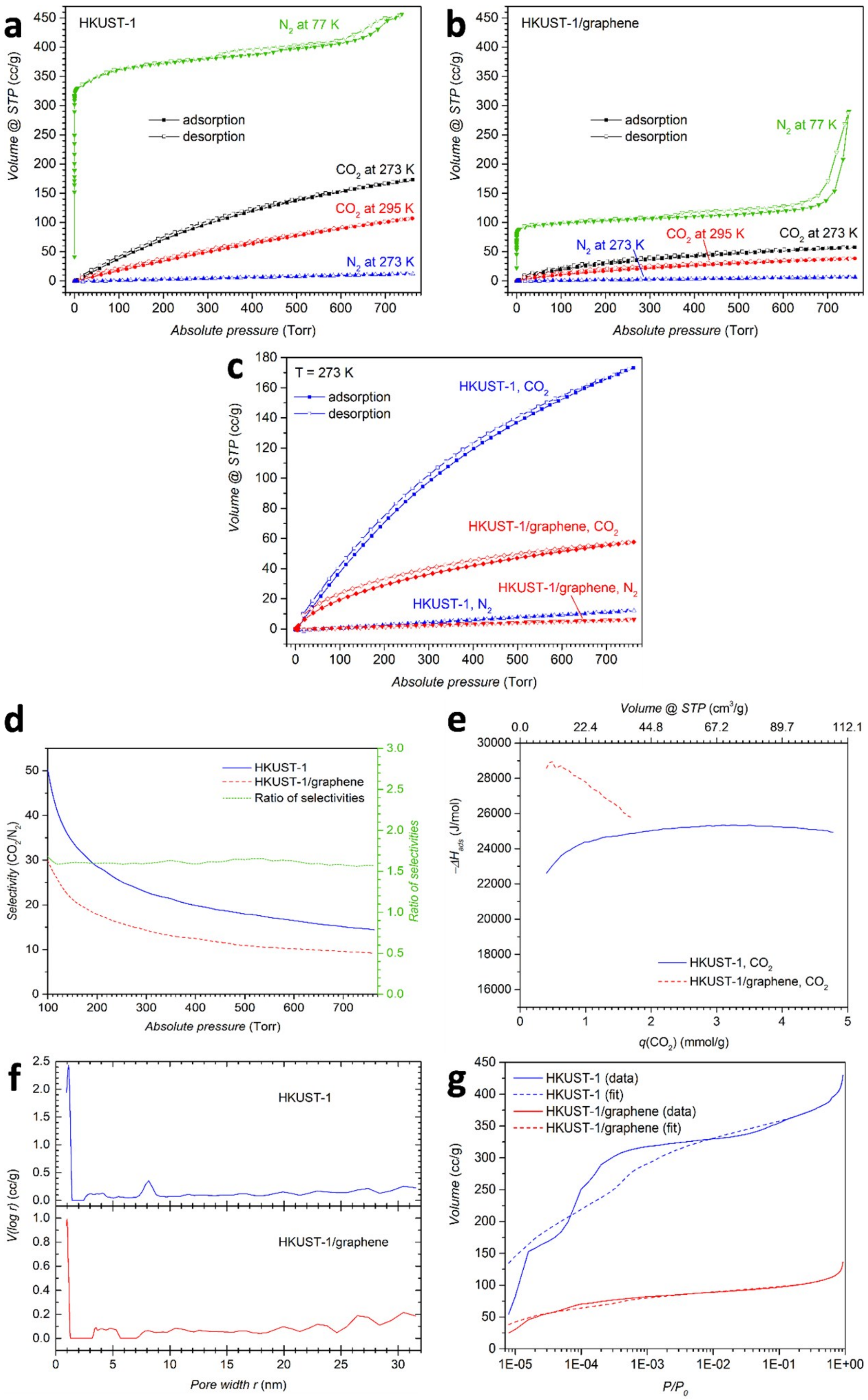
a
HKUST-1
N2 at 77 K
adsorption
desorption
CO2 at 273 K
CO2 at 295 K
N2 at 273 K
Volume @ STP (cc/g)
Absolute pressure (Torr)
b
HKUST-1/graphene
adsorption
desorption
N2 at 77 K
CO2 at 295 K
CO2 at 273 K
N2 at 273 K
Volume @ STP (cc/g)
Absolute pressure (Torr)
c
T = 273 K
adsorption
desorption
HKUST-1, CO2
HKUST-1/graphene, CO2
HKUST-1/graphene, N2
HKUST-1, N2
Volume @ STP (cc/g)
Absolute pressure (Torr)
d
HKUST-1
HKUST-1/graphene
Ratio of selectivities
Selectivity (CO2/N2)
Ratio of selectivities
Absolute pressure (Torr)
e
Volume @ STP (cm3/g)
−ΔHads (J/mol)
HKUST-1, CO2
HKUST-1/graphene, CO2
q(CO2) (mmol/g)
f
HKUST-1
HKUST-1/graphene
V(log r) (cc/g)
Pore width r (nm)
g
HKUST-1 (data)
HKUST-1 (fit)
HKUST-1/graphene (data)
HKUST-1/graphene (fit)
Volume (cc/g)
P/P0

Fig. S10. CO_2 and N_2 adsorption/desorption curves at various temperatures for bulk powders of (a) HKUST-1 and (b) HKUST-1/graphene. From the N_2-sorption curves at 77 K, it is possible to distinguish two different features of the samples: HKUST-1 resembles a type I+IV isotherm (type I dominates), whereas HKUST-1/graphene shows a type I+II isotherm. Type I and type IV curves indicate the presence of micropores and mesopores, respectively.[4, 5] We suggest that changed porosity of the composite can be ascribed to formation of pores at the interface between HKUST-1 and graphene due to graphene flakes wrapping and covering the outer surface of the MOF crystals. Moreover, type II behaviour may partly be ascribed to graphite, which was observed to be present in the bulk powder, or to the formation of macropores at the graphene/HKUST-1 interfaces;[6] however, presence of macropores has not been evidenced. The BET surface area of the composite material is much lower in comparison with the parent MOF. (c, d) Selectivity of CO_2 over N_2 adsorption at 273 K showing that CO_2 is absorbed preferentially in the two materials, and the selectivity of HKUST-1 is 1.5 times higher than the composite throughout the covered pressure range. Data below 100 Torr in (d) were excluded due to uncertainties associated with the low N_2 adsorption observed in that range. (e) Heat of adsorption of CO_2. HKUST-1/graphene binds CO_2 molecules more strongly than HKUST-1, as determined by a higher heat of adsorption (ΔH_{ads} = –29 kJ mol^{-1} for the composite and –23 kJ mol^{-1} for HKUST-1 at q = 0.5 mmol g^{-1}). The larger heat of adsorption of the composite may be due to stronger interaction between hydrophobic graphene sheets and non-polar CO_2 molecules or differences in the microstructure at the HKUST-1/graphene interface. Heat of adsorption values ΔH_{ads} for CO_2 were determined based on the isotherms measured at 273 K and 295 K, by using the Clausius-Clapeyron equation:[7] $(\ln p)_q = (\Delta H_{ads}/R)(1/T) + C$, where q is the amount of CO_2 adsorbed (mmol g^{-1}) at 273 K or 295 K, p is the absolute pressure (bar), T is the absolute temperature (K), and R is the ideal gas constant (J mol^{-1} K^{-1})). For the composite there is a trend showing that the heat of adsorption depends on the CO_2 loading: Increasing CO_2 amounts are bound less strongly in the composite. For comparison, HKUST-1 overall shows a constant heat of adsorption in the whole pressure range. This behaviour agrees well with a similar trend observed for a HKUST-1/carbon nanotube composite.[7] Fig. (f) shows the pore size distribution calculated by using built-in DFT models in the gas sorption software AsiQWin. As it can be seen in (g), the curves fitted to the experimental data do not show a perfect match, due to the fact that the applied DFT model for zeolites is not ideal for describing MOFs. In qualitative terms, the pore size distribution (f) shows that the composite retains the microporosity of the parent HKUST-1, and additionally the formation of mesopores is hinted.

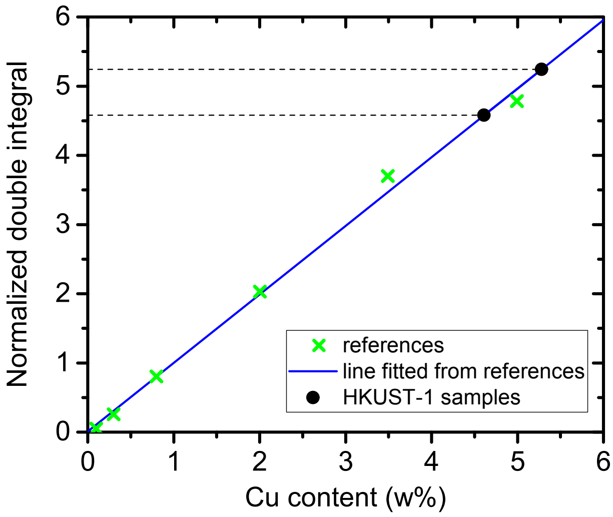

Fig. S11. Quantification of EPR signal by comparison with Cu^{2+} reference samples. Six solid reference samples were prepared by grinding known amounts of $CuSO_4{\cdot}5H_2O$ with K_2SO_4. The room temperature EPR spectra of the reference samples and of the HKUST-1 and HKUST-1/graphene powder samples were collected under identical settings of the spectrometer and with identical volume of the sample. Each spectrum was averaged over 3 sweeps. The quality-factor (Q) of the cavity was checked and was found to be constant. The spectra were corrected by subtracting the background obtained on pure K_2SO_4 and was corrected by a linear background. The double integrals of the spectra of the reference samples were calculated using MATLAB, and a line was fitted to the results. The spin concentration of the unknown samples was extrapolated by comparing the value of the double integral with the fitted line.

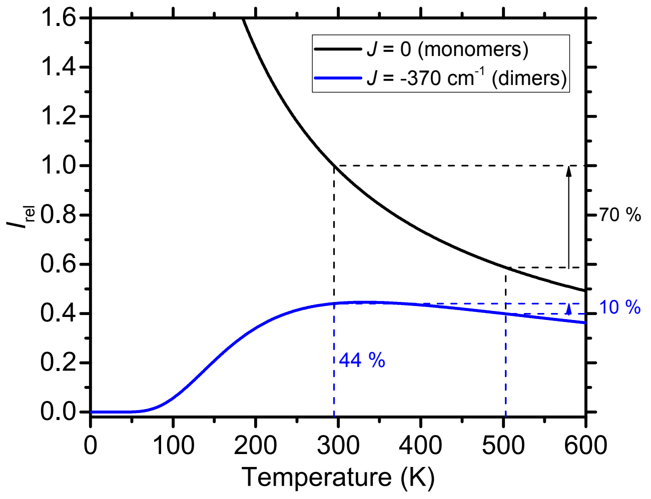

Fig. S12. Magnetic susceptibility of monomeric and dimeric paramagnetic systems. Plot of magnetic susceptibility as a function of temperature calculated using the Bleaney-Bowers equation for non-coupled *S* = ½ centres (monomers) in black and for an anti-ferromagnetically coupled dimer of S = ½ centres with a coupling constant of *J* = -370 cm^{-1} in blue. The values are given relative to the value for the monomer at room temperature. The predicted value for the dimer is 44% of the value for the monomer at room temperature. The expected increase in EPR signal upon decreasing the temperature from 230 to 22 °C (503 to 295 K) are given in % for both curves.

The EPR signal intensity is proportional to the population in the initial state of the excitation for systems where relaxation of the excited state through another pathway is not dominant. This gives the total intensity as the difference in population between the ground and excited spin state. The populations can be calculated in the same way as it is usually done for the magnetic susceptibility and in fact the EPR signal intensity can also be calculated using the Bleaney-Bowers equation for the molar magnetic susceptibility, χ_m of a dimer consisting of two *S* = ½ centres:

$$\chi_m = \mu_0 \frac{2\mu_B^2 g^2}{kT\left(3 + e^{-\frac{J}{kT}}\right)}$$

Where μ_0 is the permeability of vacuum, μ_B is the Bohr magneton, *g* is the isotropic g-value, *k* is the Boltzmann constant, and *J* is the coupling constant for the system following the Hamiltonian **H** = -$J\mathbf{S}_1 \cdot \mathbf{S}_2$ for the exchange interaction. For *J* = 0 there is no interaction between the centres and the equation simplifies to the Curie equation relevant for paramagnetic monomers.

Only the relative intensities have importance here and therefore the values calculated in Fig. S12 are given

relative to the value at 295 K (22 °C) calculated for $J = 0$.

Extended description of EPR data (Fig. 6) upon *in situ* dehydration

The following description contains technical details in addition to those presented in the manuscript:

Upon dehydration, the spectrum becomes broad and completely symmetric (Figure 6a-b). The total intensity obtained by double integration first drops moderately, then increases and stabilizes at a value, which is slightly higher than the start value for HKUST-1 and lower than the start value for HKUST-1/graphene. After the temperature decrease to room temperature, the spectra do not change shape. The intensity increases to a value which is 47(3)% higher at 22 °C compared to the value at 230 °C for both samples. The intensity of a monomeric $S = ½$ centre is expected to increase 70% with a temperature change from 230 °C to 22 °C due to the Boltzmann distribution.

The EPR signal intensity follows the magnetic susceptibility and thus depends on the magnetic coupling constant, J for a magnetically coupled dimer. For copper paddle-wheel dimers in HKUST-1, J ($\mathbf{H} = -J\mathbf{S_1}\cdot\mathbf{S_2}$) is found to be -370 cm^{-1}.[8] This value corresponds to an expected intensity of the coupled dimer of 44% compared to the intensity of the analogous isolated monomers. The temperature dependence of the theoretical curve gives an expected increase in EPR signal intensity of only 10% with a temperature change from 230 °C to 22 °C (Figure S12). The intensity gain is 47(3)% which is between the two limiting situations, *i.e.* 70% for pure monomeric copper and 10% for pure dimeric copper. Therefore, again both monomeric and dimeric copper species are suggested to be present in both samples. HKUST-1 started with EPR signal corresponding to 5.2 wt% copper under the assumption of 100% monomers obeying the Curie law. After dehydration, this increases to 7.64 wt% for HKUST-1. If we instead assume 100% strongly coupled paddle-wheel dimers ($J = -370$ cm^{-1}) we have accounted for 17 wt% of the copper after dehydration. Either limiting situation leaves a significant amount of the copper EPR signal not accounted for compared to the 29 wt% Cu in pure HKUST-1. Some of the missing signal may have been lost due to long range interactions between paramagnetic centres, but the presence of EPR inactive copper cannot be excluded.

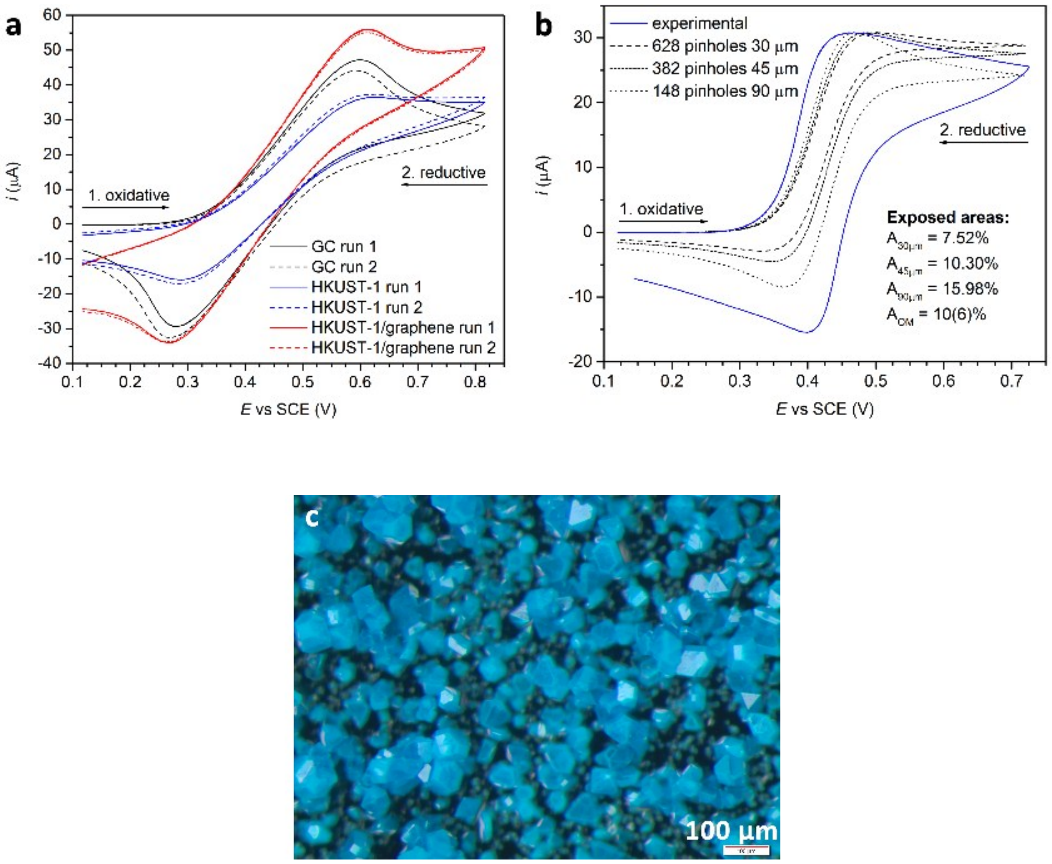

Fig. S13. (a) Voltammograms collected for a bare GC electrode and for HKUST-1- and composite-coated electrodes, using circular masks with an aperture of approximately 2.7 mm in diameter (voltammograms of 2 mM ferrocene (Fc) recorded in 0.01 M TBA·BF_4/acetonitrile, sweep rate = 0.1 V s^{-1}). No baseline correction or iR compensation was applied while collecting these data. The large slope of the composite sample might be due to intercalated species (*e.g.* solvent or TBA·BF_4), but this was not further investigated. (b) Simulated curves resulting from fits to the experimental oxidation peak of the HKUST-1-covered electrode. The *DigiSim®* software was used to run the simulations. The mismatch between the experimental reduction wave and the simulated ones is due to the fact that capacitive current contributions were not accounted for in the software. Cyclic voltammograms were simulated based on pinholes sizes of 30 µm and 45 µm, respectively. These sizes correspond to the approximate exposed GC areas according to an optical microscopy (OM) image (c) of the HKUST-1 covered electrode. Additionally, a simulation was carried out by using an ensemble of bigger pinholes (90 µm). This simulation takes into account that the exposed GC areas may be ascribed to smaller pinholes as well as defected zones with larger uncoated areas of GC.

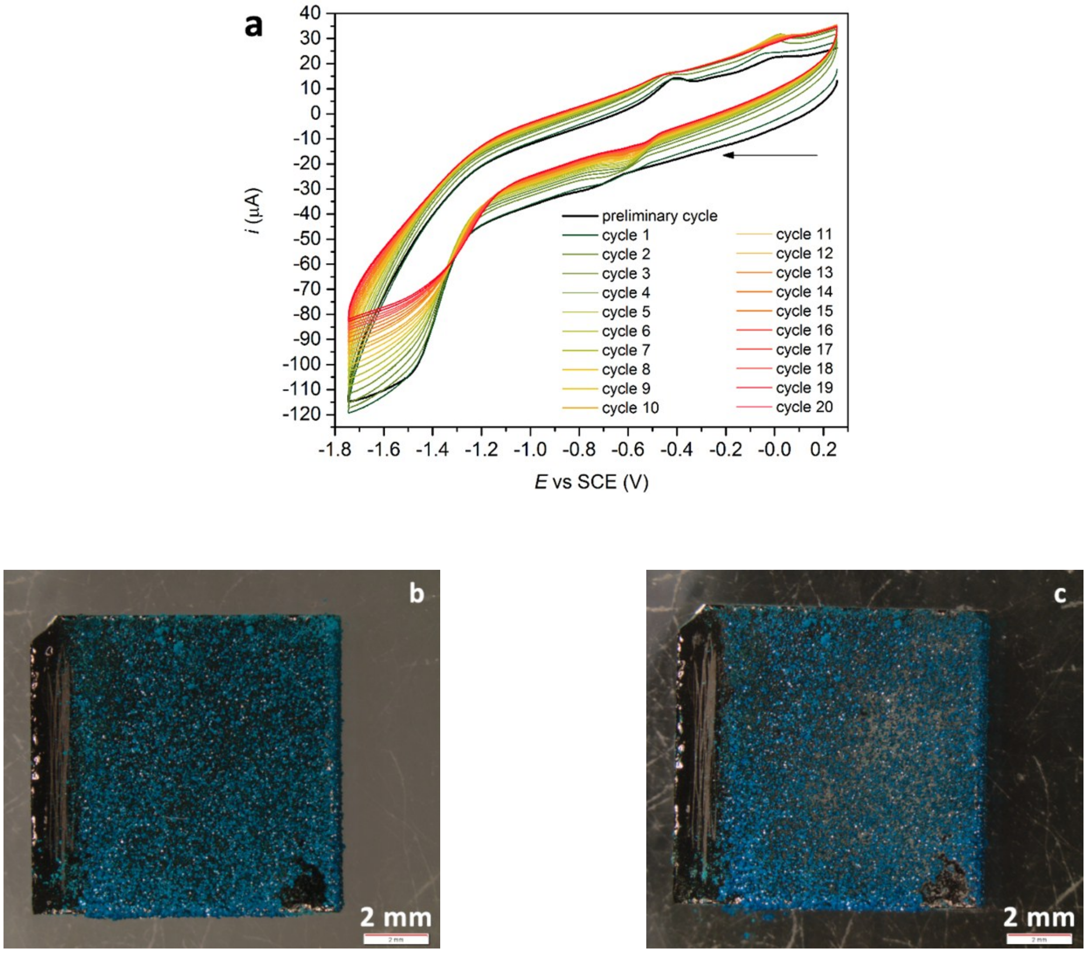

Fig. S14. (a) Voltammograms representing 20 subsequent cycles recorded in argon without stirring (from *green* to *red*) using an HKUST-1/graphene film covered GC substrate. A preliminary curve (black) and the subsequent curve (green) are nearly coinciding. This shows that upon stirring the current response of the electrode is substantially unchanged due to mixing of the solution and regeneration of a diffusion layer. On the other hand, looking at the series of 20 measurements without stirring, a decrease in current is observed due to the gradual pauperisation of the electrolyte in the diffusion layer. The arrow indicates the order in which the CVs were collected. The voltammograms were recorded in 0.01 M TBA·BF_4/acetonitrile, sweep rate = 0.050 V s^{-1}. Optical microscopy images showing the HKUST-1/graphene film before (b) and after (c) 20 CV cycles in Ar-saturated 0.01 M TBA·BF_4/acetontrile. The photos indicate film stability, as no material appears to be lost. The appearance of a lighter phase after CV is due to deposition of TBA·BF_4 from the electrolyte solution on the GC surface. Please note, that the plate was one of the first attempts to grow composite films, which is the reason for the incomplete coverage.

a HKUST-1 (no treatment)

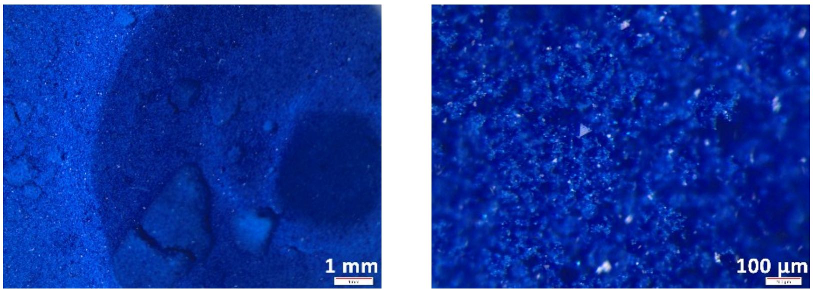

b Annealing at 230 °C

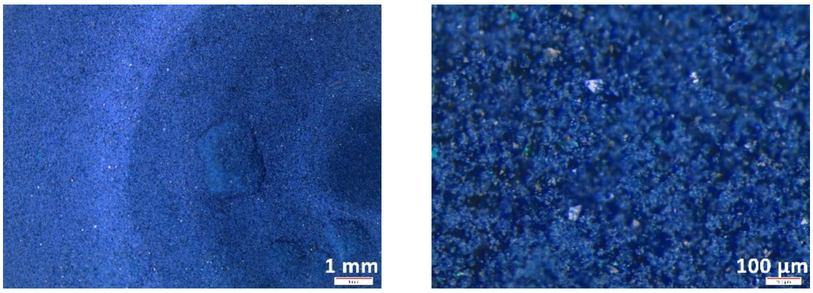

c Soaking in 0.5 M $KHCO_3/H_2O$

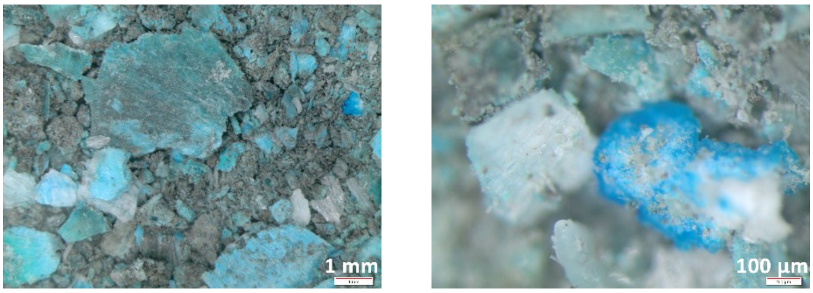

d Soaking in 0.5 M H_2SO_4/H_2O

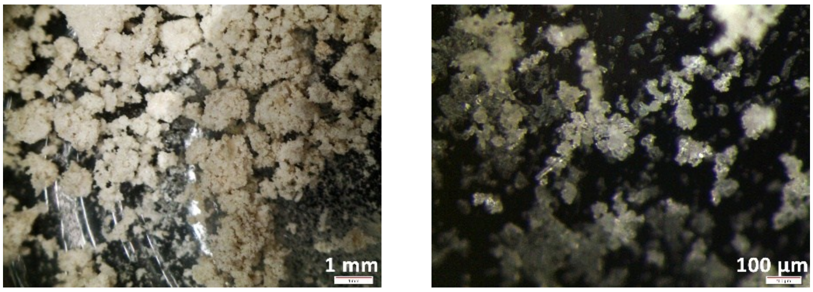

e Soaking in 0.01 M

TBA·BF_4/DMF

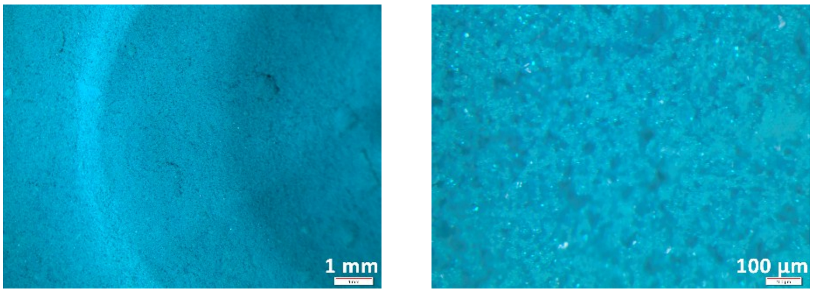

f Soaking in 0.01 M

TBA·BF_4/acetonitrile

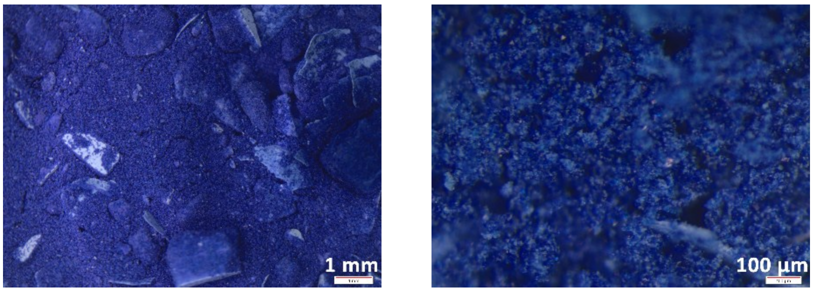

Fig. S15. Optical microscopy images at different magnifications showing the stability of HKUST-1 crystals upon chemical treatment and subsequent drying at 70 °C. From top to bottom, we observed the following: (a) Untreated HKUST-1 forms regular octahedral blue crystals. (b) Annealing at 230 °C does not seem to damage the crystals. (c) Soaking the MOF in 0.5 M $KHCO_3$/H_2O destroys the material in less than a minute and forms various impurities. (d) HKUST-1 is rapidly destroyed in 0.5 M H_2SO_4/H_2O and white crystals form as a precipitate. In contrast, the solution turns blue, evidencing Cu(II) in solution. (e) HKUST-1 soaked in 0.01 M TBA·BF_4/DMF does not affect the morphology of the crystals. However, a solvatochromic effect is observed, with the powders becoming greenish, which may be due to DMF residues in the pores. (f) Soaking the material in 0.01 M TBA·BF_4/acetonitrile (ACN) does not change the structure of HKUST-1. PXRD patterns of the samples (a)-(f) are shown in Fig. S16.

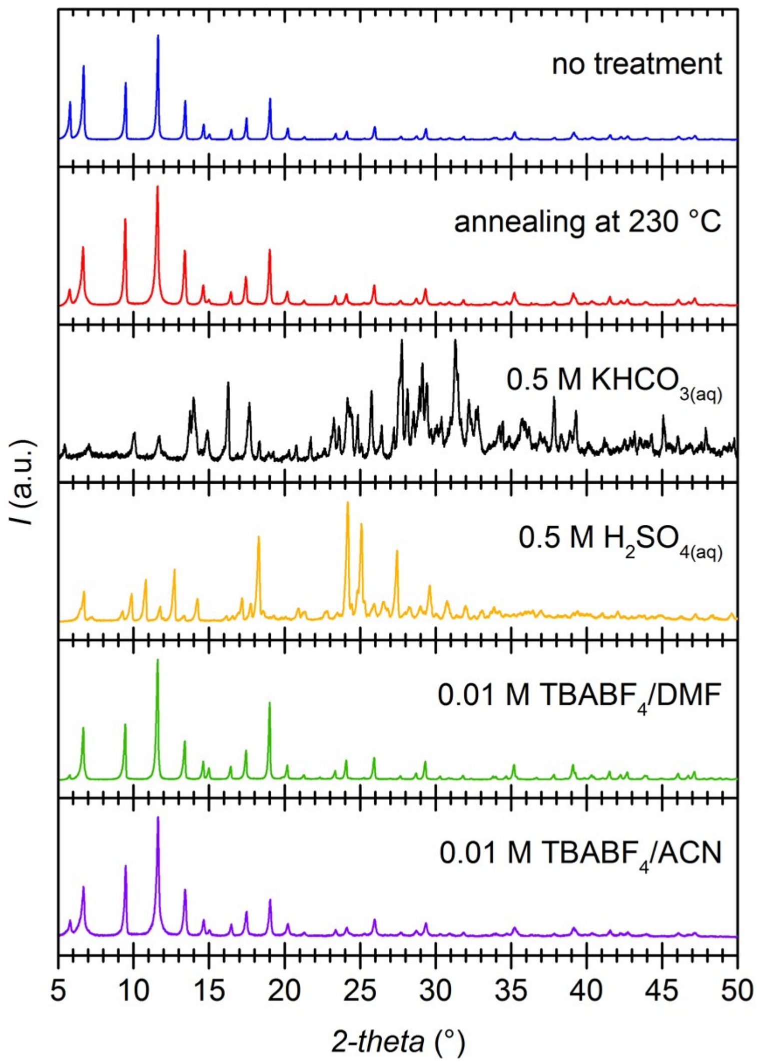

Fig. S16. PXRD data collected for the powder samples shown in Fig. S15 (same colour code is applied). All samples except for the one soaked in 0.5 M $KHCO_3/H_2O$ and in 0.5 M H_2SO_4/H_2O retain the HKUST-1 crystal structure.

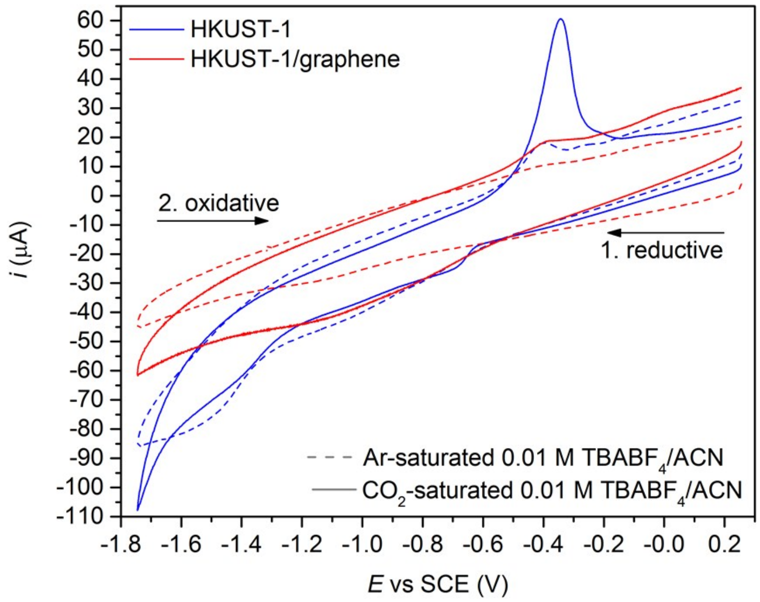

Fig. S17. CV studies do not clearly show an increase in current when the system is tested in CO_2-saturated acetonitrile (ACN) (full line) instead of an Ar-saturated (dashed line) electrolyte solution. HKUST-1 (blue) in presence of CO_2 has some characteristic peaks, which may be ascribed to redox waves at the copper centres. These signals are basically absent for the composite (red), which instead shows a weak and broad reduction band centred around –1.1 V vs. SCE. Overall, the currents are low, which suggest the material is not suitable for electrocatalytic CO_2 reduction under these conditions. The voltammograms were recorded in 0.01 M TBA·BF_4/ acetonitrile (ACN), sweep rate: 0.050 V s^{-1}.

References

1. A. C. Ferrari and D. M. Basko, *Nat. Nanotechnol.*, 2013, **8**, 235-246.
2. A. Eckmann, A. Felten, A. Mishchenko, L. Britnell, R. Krupke, K. S. Novoselov and C. Casiraghi, *Nano Lett.*, 2012, **12**, 3925-3930.
3. A. C. Ferrari, J. C. Meyer, V. Scardaci, C. Casiraghi, M. Lazzeri, F. Mauri, S. Piscanec, D. Jiang, K. S. Novoselov, S. Roth and A. K. Geim, *Phys. Rev. Lett.*, 2006, **97**, 187401-187404.
4. K. S. W. Sing, D. H. Everett, R. A. W. Haul, L. Moscou, R. A. Pierotti, J. Rouquérol and T. Siemieniewska, *Pure Appl. Chem.*, 1985, **57**, 603-619.
5. M. Thommes, K. Kaneko, A. V. Neimark, J. P. Olivier, F. Rodriguez-Reinoso, J. Rouquerol and K. S. W. Sing, *Pure Appl. Chem.*, 2015, **87**, 1051-1069.
6. E. Castillejos-Lopéz, D. M. Nevskaia, V. Muñoz, I. Rodríguez-Ramos and A. Guerrero-Ruiz, *Langmuir*, 2004, **20**, 1013-1015.
7. L. Ge, L. Wang, V. Rudolph and Z. Zhu, *RSC Adv.*, 2013, **3**, 25360-25366.
8. A. Pöppl, S. Kunz, D. Himsl and M. Hartmann, *J. Phys. Chem. C*, 2008, **112**, 2678-2684.
